# Acetyl Phosphate as a Primordial Energy Currency at the Origin of Life

**DOI:** 10.1007/s11084-018-9555-8

**Published:** 2018-03-03

**Authors:** Alexandra Whicher, Eloi Camprubi, Silvana Pinna, Barry Herschy, Nick Lane

**Affiliations:** Department of Genetics, Evolution and Environment, University College London, Darwin Building, Gower Street, London, WC1E 6BT UK

**Keywords:** Acetyl phosphate, ATP, Thioester, Phosphorylation, Metabolism, Origin of life

## Abstract

Metabolism is primed through the formation of thioesters via acetyl CoA and the phosphorylation of substrates by ATP. Prebiotic equivalents such as methyl thioacetate and acetyl phosphate have been proposed to catalyse analogous reactions at the origin of life, but their propensity to hydrolyse challenges this view. Here we show that acetyl phosphate (AcP) can be synthesised in water within minutes from thioacetate (but not methyl thioacetate) under ambient conditions. AcP is stable over hours, depending on temperature, pH and cation content, giving it an ideal poise between stability and reactivity. We show that AcP can phosphorylate nucleotide precursors such as ribose to ribose-5-phosphate and adenosine to adenosine monophosphate, at modest (~2%) yield in water, and at a range of pH. AcP can also phosphorylate ADP to ATP in water over several hours at 50 °C. But AcP did not promote polymerization of either glycine or AMP. The amino group of glycine was preferentially acetylated by AcP, especially at alkaline pH, hindering the formation of polypeptides. AMP formed small stacks of up to 7 monomers, but these did not polymerise in the presence of AcP in aqueous solution. We conclude that AcP can phosphorylate biologically meaningful substrates in a manner analogous to ATP, promoting the origins of metabolism, but is unlikely to have driven polymerization of macromolecules such as polypeptides or RNA in free solution. This is consistent with the idea that a period of monomer (cofactor) catalysis preceded the emergence of polymeric enzymes or ribozymes at the origin of life.

**Electronic supplementary material:**

The online version of this article (10.1007/s11084-018-9555-8) contains supplementary material, which is available to authorized users.

## Introduction

Phylogenetics and comparative physiology suggest that the earliest cells were autotrophic (Woese 1977; Baross and Hoffman 1985; Morowitz et al. 2000; Martin and Russell 2003; Smith and Morowitz 2004; Russell and Martin 2004; Braakman and Smith 2012; Weiss et al. 2016), living from relatively unreactive gases such as H_2_ and CO_2_, arguably via the acetyl CoA pathway (Russell and Martin 2004; Martin and Russell 2007; Sousa et al. 2013; Martin et al. 2014; Sojo et al. 2016). This pathway is the only CO_2_-fixation pathway found in both bacteria and archaea (Russell and Martin 2004; Martin and Russell 2007; Fuchs 2011). It is short, linear, and exergonic (Sousa et al. 2013; Martin et al. 2014) generating acetyl-CoA from H_2_ and CO_2_ via enzymes that contain multiple Fe(Ni)S clusters with structures similar to minerals such as greigite (Russell and Martin 2004; Kim et al. 2013; Harel et al. 2014; White et al. 2015). From acetyl CoA, a series of analogous hydrogenation and carbonylation reactions can produce carboxylic acids (Krebs cycle intermediates) from pyruvate to isocitrate (Camprubi et al. 2017; Muchowska et al. 2017). These are the precursors of amino acid, nucleotide, carbohydrate and lipid synthesis in essentially all living cells (Smith and Morowitz 2004, 2016; Martin and Russell 2007). But carboxylic acids are also tardily reactive as a starting point for these biosyntheses. Accordingly, metabolism is typically primed through formation of thioesters or phosphorylation by ATP (Srinivasan and Morowitz 2009). That poses a paradox at the origin of life as the synthesis of monomers such as nucleotides from H_2_ and CO_2_ via Krebs cycle intermediates has so far proved intractable under prebiotic conditions, despite its theoretical appeal.

In contrast, prebiotic syntheses of complex monomers such as activated nucleotides have been successful when starting from relatively reactive substrates such as cyanide, cyanoacetylene or formamide, typically energized by UV radiation (Powner et al. 2009; Saladino et al. 2012; Patel et al. 2015) or even proton irradiation (Saladino et al. 2015). While this is impressive synthetic chemistry, its relevance is ambiguous, as these syntheses do not resemble the biochemistry of extant cells in terms of substrates, catalysts, reaction pathways or energy coupling (Sojo et al. 2016; Keller et al. 2017a). Cyanide is not used by living cells as a source of either carbon or nitrogen for metabolism, nor is UV (or proton) radiation used as an energy source (Lane et al. 2010). Putatively prebiotic phosphorylating agents such as diamidophosphate are never used in modern cells and act by distinct mechanisms, primarily phosphorylating amino groups (Gibard et al. 2017). Similarly, meteoritic phosphate minerals such as schreibersite can phosphorylate nucleotides, but cannot be homologous to modern cells (Gull et al. 2015). If prebiotic chemistry really was so dissimilar to modern biochemistry, then at some point cells must have adopted radically different metabolic chemistry, starting with CO_2_ and promoting tardy reactions using ATP. If so, that exposes a severe and unexplained discontinuity at the origin of life. Whichever way the problem is seen, then, the emergence of biochemistry from geochemistry remains an unsolved problem.

Like all autotrophs, cells that use the acetyl CoA pathway depend on electrochemical ion gradients across membranes to drive CO_2_ fixation (Buckel and Thauer 2013). Specifically, methanogens use flavin-based electron bifurcation to generate a membrane potential (Kaster et al. 2011; Wagner et al. 2017) which is then used to reduce ferredoxin via the energy-converting [NiFe] hydrogenase Ech (Hedderich 2004; Thauer et al. 2008; Buckel and Thauer 2013). This is arguably the simplest and most direct use of membrane potential, hence could give insight into a prebiotic mechanism of CO_2_ fixation. We have argued that geologically sustained proton gradients could have modulated the reduction potential of both H_2_ and CO_2_ in alkaline hydrothermal systems, facilitating their reaction across semiconducting FeS barriers (Lane 2014; Herschy et al. 2014; Sojo et al. 2016) or fatty-acid membranes containing associated FeS crystals (West et al. 2017) to form small organics, notably Krebs cycle intermediates (Camprubi et al. 2017) in a manner analogous to Ech in methanogens (Sojo et al. 2016). Ancient alkaline hydrothermal systems provided H_2_, CO_2_, FeS barriers and geochemically sustained proton gradients (Russell et al. 1994; Russell and Hall 1997; Martin and Russell 2003, 2007; Sousa et al. 2013; Martin et al. 2014), conditions that hypothetically segue into CO_2_ reduction by H_2_ via a homologous proton-motive proto-Ech in the first cells (Sojo et al. 2016; West et al. 2017). Yet while vectorial prebiotic chemistry could feasibly form Krebs cycle intermediates (Camprubi et al. 2017), it is unlikely to have driven further intermediary metabolism in the absence of equivalents to acetyl CoA or ATP.

**Fig. 1:**
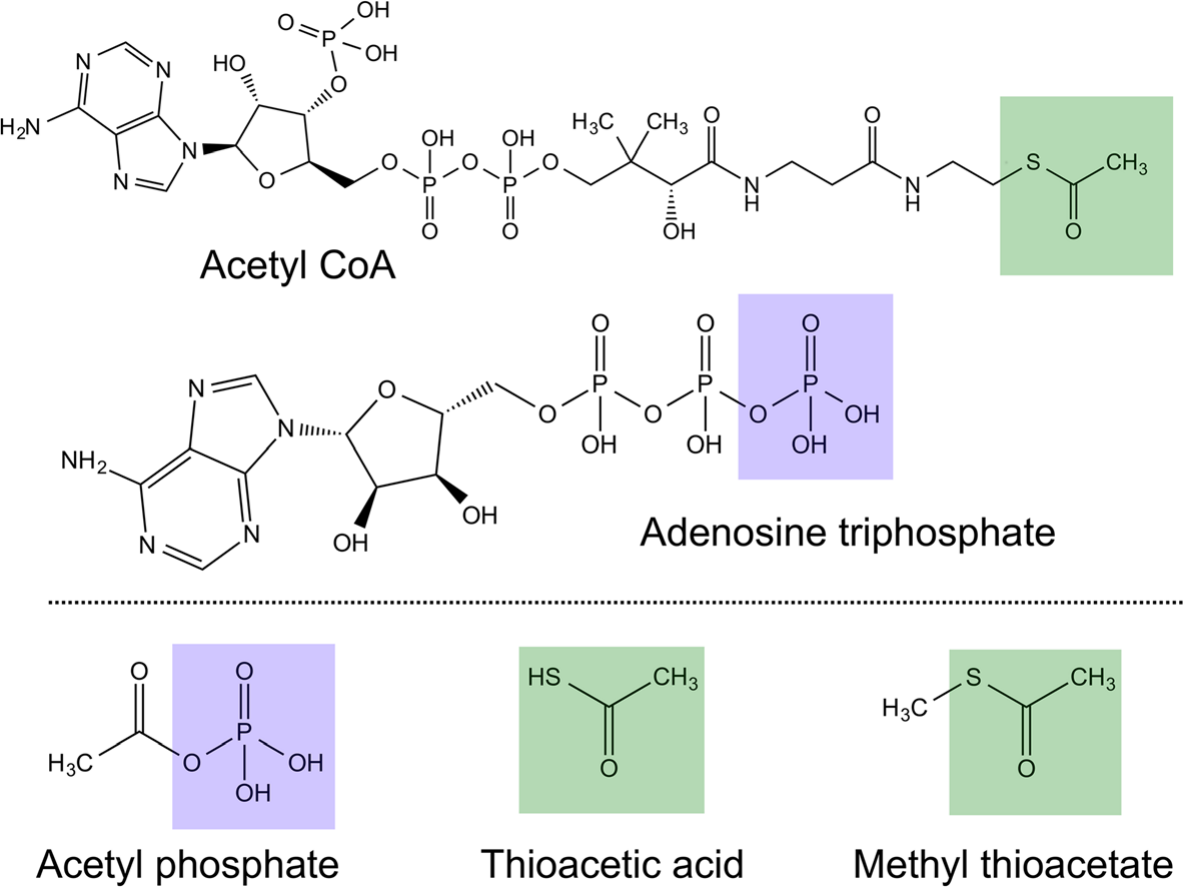
Molecular structures of key biotic and prebiotic molecules. Acetyl CoA is the hub of metabolism in all six known pathways of carbon fixation. The ‘business end’ of the molecule is the reactive thioester group (green shaded section), which has prebiotic analogues in methyl thioacetate and thioacetic acid. Adenosine triphosphate (ATP) is the universally conserved energy currency in modern metabolism; acetyl phosphate (AcP) could have been a simple precursor, driving similar reactions. The blue shading highlights equivalent phosphate groups

Both acetyl CoA and ATP are universally conserved across life, hence are most probably ancient (Morowitz et al. 2000; Smith and Morowitz 2004; Fuchs 2011; Martin et al. 2014). Nonetheless, both are complex molecules produced by genetically encoded enzymes, and so are unlikely to have driven the emergence of biochemistry at the origin of life. Plausible prebiotic precursors to acetyl CoA and ATP have been proposed to operate in a ‘thioester’ world (de Duve 1988, 1991; 1998; Sousa et al. 2013; Goldford et al. 2017). Prebiotic thioesters such as methyl thioacetate could arguably phosphorolyse to generate a phosphoester bond equivalent to that in ATP, as in the simple 2-carbon molecule acetyl phosphate, AcP (Ferry and House 2006; Martin et al. 2014; Sojo et al. 2016). This is analogous to modern cells, where acetyl-CoA is readily phosphorolysed to generate AcP in both bacteria and archaea (Decker et al. 1970; Thauer et al. 1977; Martin and Russell 2007). AcP then phosphorylates ADP to ATP (Ferry and House 2006; Schönheit et al. 2016) making it the fulcrum between thioester and phosphate metabolism (de Duve 1991). In terms of prebiotic chemistry, AcP could have been formed from simple thioesters such as methyl thioester, and then driven phosphorylation and condensation reactions in a similar fashion to ATP (Fig. 1) (de Duve 1991; Ferry and House 2006; Martin and Russell 2007), narrowing the gap between geochemistry and the origins of intermediary metabolism.

However, simple thioesters such as methyl thioacetate and putative ATP analogues like AcP have generally been considered to be too unstable to persist for long enough to drive prebiotic chemistry, especially at the high or low pH and hot temperatures found in hydrothermal systems (Barge et al. 2014; Chandru et al. 2016). AcP has long been overlooked experimentally as a prebiotic precursor to ATP, except in the synthesis of more stable (but less reactive) products such as pyrophosphate (Baltscheffsy and Baltscheffsky 1995; Kornberg et al. 1999; de Zwart et al. 2004; Barge et al. 2014)*.* This perception stems largely from the ‘heterotrophic’ approach to prebiotic chemistry: stable organics are assumed to accumulate in a primordial soup or warm geothermal pond, perhaps with different precursors formed under distinct conditions before being brought together by some circumstance (Patel et al. 2015), all of which demands long-term stability. In contrast, autotrophic origins imply a continuous flux of inorganic substrates (e.g. H_2_ and CO_2_) in a specific environment, in which organic synthesis is driven by the continuous flux, and the rates of synthesis and breakdown or reaction at least balance over time (Branscomb and Russell 2013; Lane 2015; Barge et al. 2017). It does not matter if prebiotic thioesters or AcP react within minutes or hours so long as they are formed at a similar rate; indeed it is more parsimonious if they do, as they are then approaching the lability of biochemical intermediates.

Here we examine the synthesis and reactivity of AcP under mild alkaline hydrothermal conditions with this lability in mind. We show that AcP can indeed be formed readily under ambient or mild hydrothermal conditions, has an ideal balance between stability and reactivity, and is able to drive phosphorylation (but not condensation) reactions in water, making it a plausible and biologically meaningful link between prebiotic chemistry and monomer biochemistry.

## Results

We have considered temperatures from 20 to 60 °C and pH values from 6 to 12, equivalent to the ranges found in alkaline hydrothermal vents (Russell et al. 1994, 2014; Martin et al. 2008; Nitschke and Russell 2009; Lane and Martin 2012). Such vents are composed of labyrinths of interconnected micropores with thin inorganic walls, which separate hydrothermal fluids (pH 9–12) and ocean waters (Russell et al. 1994; Russell and Hall 1997; Kelley et al. 2005). The oceans were probably mildly acidic (pH 5–7) in the Hadean, as CO_2_ levels were higher (Pinti 2005; Arndt and Nisbet 2012). Mixing within the vent is driven by convection and thermal diffusion (Braun and Libchaber 2002; Baaske et al. 2007; Mast and Braun 2010; Mast et al. 2013; Herschy et al. 2014; Kreysing et al. 2015) which is important for three reasons. First, conditions for organic synthesis are not necessarily warm and strongly alkaline, but could equally be cool and neutral pH, or even mildly acidic. Second, convective cycling means that products formed in cool, neutral conditions can cycle through warm, alkaline conditions, potentially driving other reactions, including simple hydrolysis. Third, thermal cycling can concentrate small organics such as nucleotides by at least 5000-fold via thermophoresis (Baaske et al. 2007; Mast and Braun 2010; Mast et al. 2013; Herschy et al. 2014; Kreysing et al. 2015) converting low yields (e.g. μM range) into high concentrations (e.g. high mM range) that favor molecular interactions and ultimately the polymerization of amino acids and nucleotides (Mast et al. 2013; Herschy et al. 2014). Such extreme concentration by thermophoresis partially justifies the relatively high concentrations of reagents used in this paper (see Discussion).

**Fig. 2:**
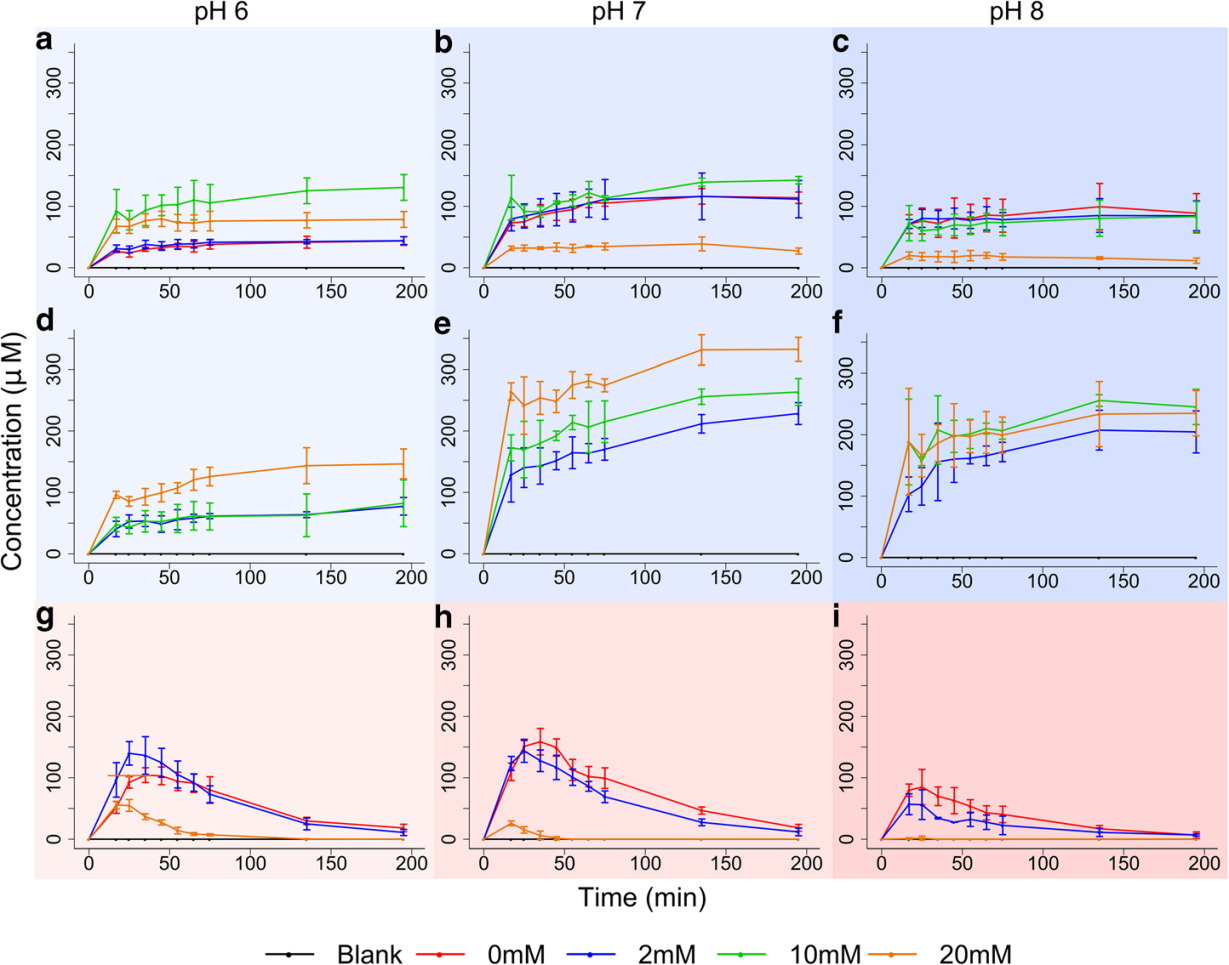
Synthesis of AcP from orthophosphate and thioacetate. **a**–**c** AcP synthesis at pH 6, 7 and 8, respectively, with varying equimolar concentrations of Ca^2+^ and Mg^2+^ ions at 20 °C. **d**–**f** varying concentrations of Mg^2+^ ions alone. **g**–**i** varying concentrations of both Ca^2+^ and Mg^2+^ ions at 50 °C. *N* = 3 ± SD

We expected to synthesise AcP by phosphorolysing methyl thioacetate (CH_3_COSCH_3_). This simple prebiotic thioester has been argued to have properties equivalent to acetyl CoA (de Duve 1988, 1991; Martin and Russell 2007; Lane and Martin 2012), and has indeed been formed under hydrothermal conditions from CO and CH_3_SH alone (Huber and Wächtershäuser 1997). In fact, we did not detect any AcP from CH_3_COSCH_3_ in the presence of 20 mM Na_2_HPO_4_, but rather generated yields of up to 2% within 1–2 h from the even simpler precursor thioacetate (CH_3_COSH) (Figs. 1 and 2). The yield depended on pH, temperature, and ions present. Under mildly acidic conditions and cooler temperatures (pH 6, 20 °C), equimolar mixtures of Ca^2+^ and Mg^2+^ ions promoted synthesis of AcP, compared with no ions (Fig. 2a). Under more alkaline conditions, Ca^2+^ and Mg^2+^ ions lowered AcP synthesis, because Ca^2+^ precipitated out some phosphate as apatite, so less was available for AcP formation (Fig. 2b and c; data for Ca^2+^ alone are not shown, as nanoparticles interfered with NMR measurements). AcP was not formed at all at pH 11, even in the absence of ions (Online resource 1). In contrast, Mg^2+^ alone precipitated relatively little phosphate below pH 11, and doubled yields of AcP under neutral or mildly alkaline conditions, presumably promoting synthesis relative to breakdown of AcP (Fig. 2d–f). These data foreshadow the close association of Mg^2+^ ions with AcP, ATP and nucleotides, and the exclusion of Ca^2+^ ions from modern cells. Synthesis under anaerobic conditions did not affect the yield of AcP, indicating that thioacetate is equally reactive in the absence of oxygen (Online resource 2). Addition of Fe^2+^ also had little effect on AcP synthesis when the experiments were repeated under anoxic conditions (Online resource 2).

**Fig. 3:**
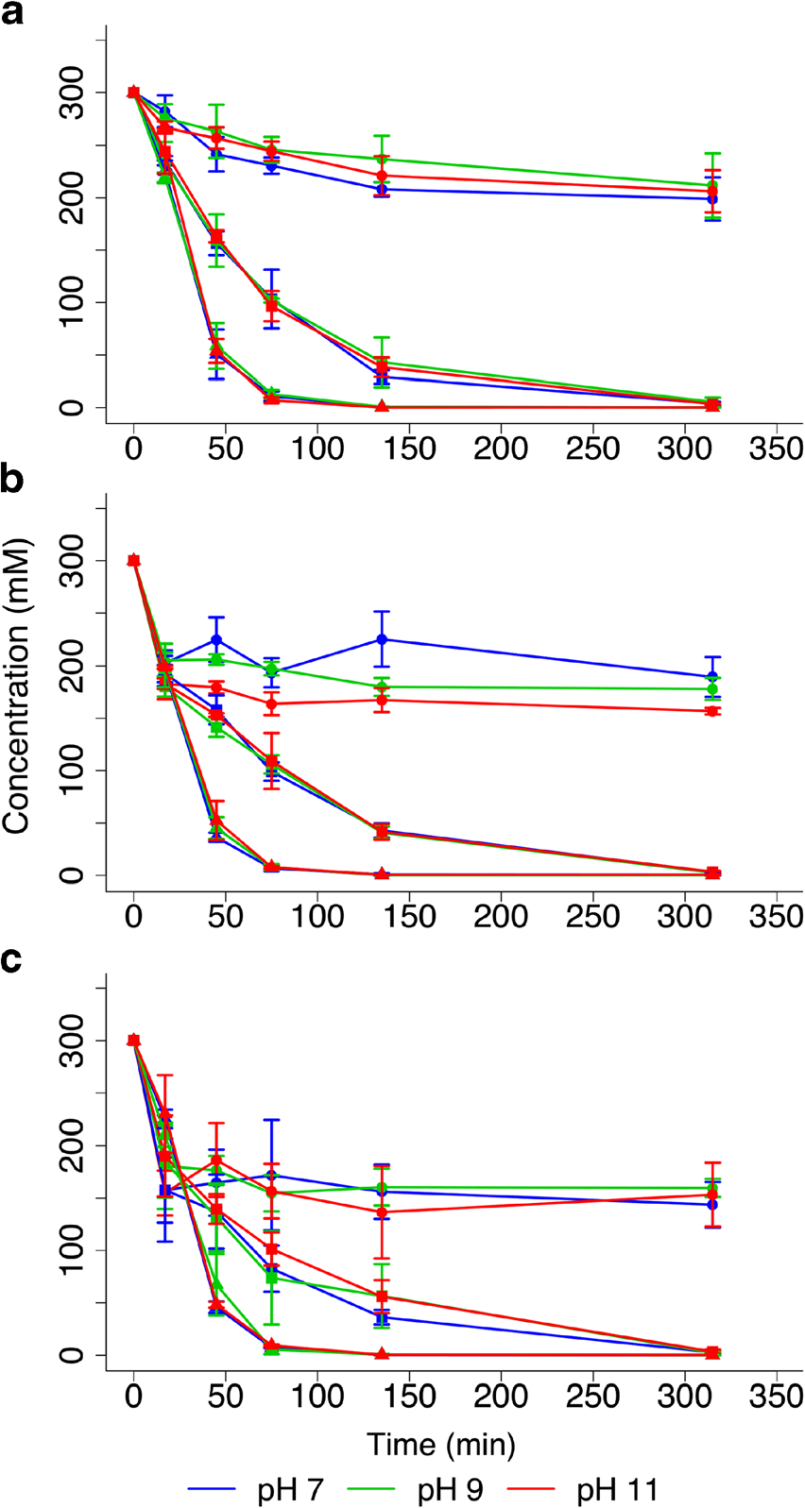
Stability of AcP depending on pH, temperature and ions. Degradation profile for AcP over 5 h with and without ions at pH 7, 9 and 11, stored at 20 °C (circles), 50 °C (squares) and 60 °C (triangles). **a** no ions added, **b** with 20 mM Mg^2+^ ions added, **c** with 20 mM Ca^2+^ ions added. N = 3 ± SD

At ambient temperatures and non-extreme pH, AcP is reasonably stable, as previously reported by others (Koshland 1952; Etaix and Buvet 1975) with ~20% hydrolysed over 5 h at 20 °C under the range of pH conditions tested (Fig. 3a). The rate of hydrolysis depends strongly on temperature, with AcP completely hydrolysed within 3–5 h at 50 °C, and within 90 min at 60 °C (Fig. 3a). In contrast, pH had little effect at any temperature (Fig. 3a). The presence of Mg^2+^ (Fig. 3b) and Ca^2+^ (Fig. 3c) ions also had little effect, slightly speeding the initial rate of hydrolysis at both 20 °C and 50 °C, as reported previously for concentrated salt solutions by Di Sabato and Jencks (1961b), but not changing the overall proportion hydrolysed over 5 h (Fig. 3b and c). However, at higher temperatures (50 °C), the balance between AcP formation and hydrolysis shifted markedly towards hydrolysis (Fig. 2g–i). Although the initial rate of synthesis was slightly faster, AcP was almost completely hydrolysed within 3 h, especially under mildly alkaline conditions (pH 8; Fig. 2i). In the vent setting, therefore, AcP synthesis should occur mostly in cooler, more neutral regions, precisely the regions where organics tend to accumulate by thermophoresis (Baaske et al. 2007; Herschy et al. 2014), and should be stable under aqueous neutral to alkaline conditions over at least several hours.

**Fig. 4:**
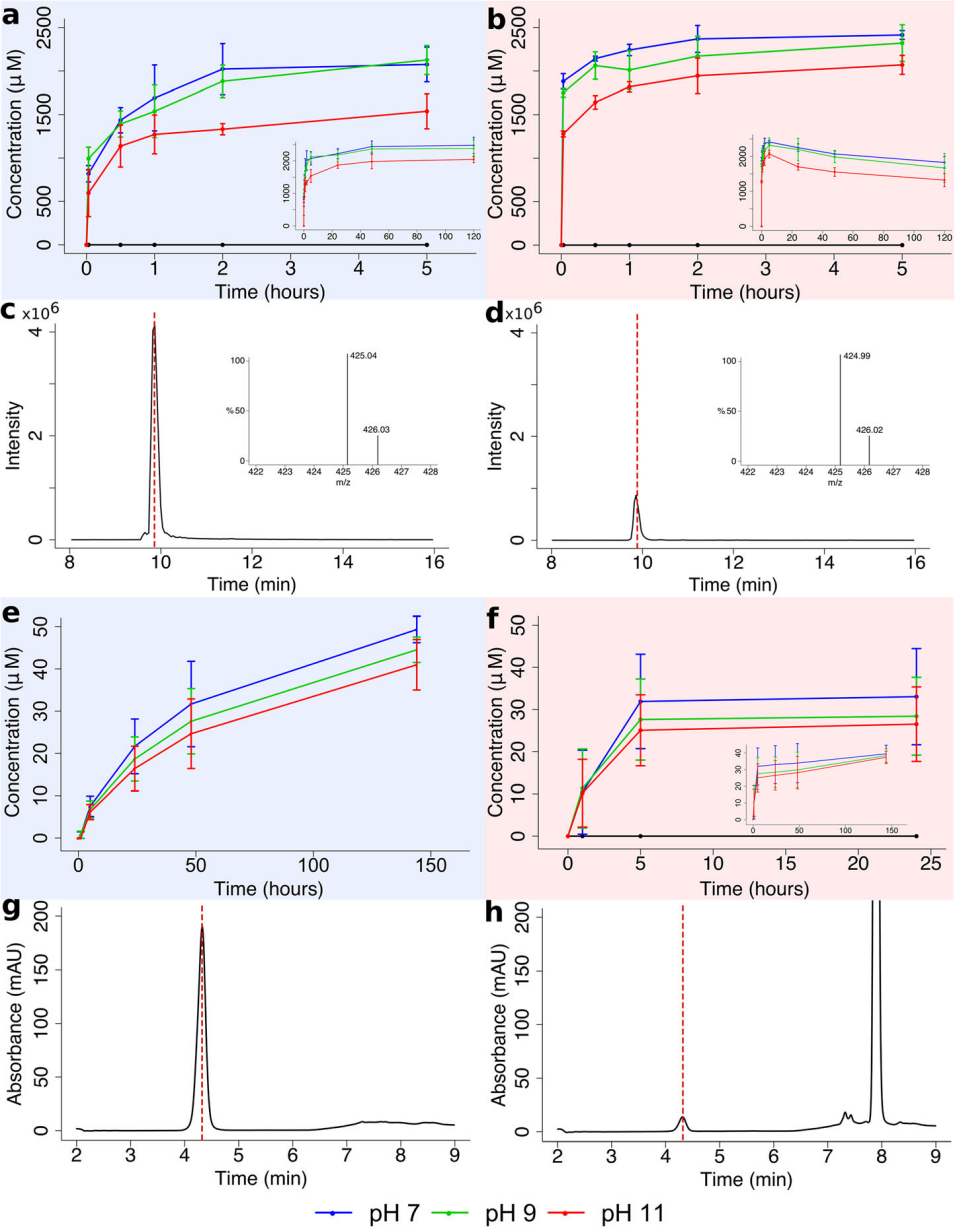
Phosphorylation of ribose and adenosine by AcP. **a**–**b** Synthesis of ribose-5-phosphate from D-ribose and AcP at pH 7, 9 and 11 at: **a** 20 °C and (**b**) 50 °C. Graph inserts show full reaction profile over 120 h. **c**–**d** LC-MS chromatogram for derivatised ribose-5-phosphate (monoisotopic mass: 425.14 m/z): **c** standard and (**d**) experimental sample. Graph inserts show mass spectra for each peak. **e**–**f** Phosphorylation of adenosine to adenosine monophosphate (AMP) by AcP at pH 7, 9 and 11: **e** 20 °C and (**f**) 50 °C. Graph insert shows full reaction profile over 144 h. **g**–**h** HPLC-UV chromatogram for detection of AMP at 4.3 min at 254 nm UV wavelength; **g** standard and (**h**) experimental sample. The large peak at 7.9 min is adenosine. N = 3 ± SD

Once formed, AcP can indeed phosphorylate substrates. We considered first whether AcP could phosphorylate nucleotide precursors under a range of alkaline hydrothermal conditions. Given their symbolic relevance to RNA synthesis, we specifically examined the synthesis of ribose-5-phosphate (R5P) from ribose, adenosine monophosphate (AMP) from adenosine, and ATP from ADP. For R5P, the overall yield (relative to ribose) was modest (~2%) but the rate of synthesis was rapid, with >500 μM R5P formed within 8 min at 20 °C in the absence of Ca^2+^ and Mg^2+^ ions, and concentrations peaking at ~2500 μM after 120 h (Fig. 4a, c–d). R5P is surprisingly stable at 20 °C, with no loss of yield over 5 days (Fig. 4a inset). The rate of synthesis of R5P was even faster at 50 °C, with concentrations reaching ~1500 μM within 8 min and ~2300 μM after 2 h, giving a similar total yield (Fig. 4b). However, ~20% of this yield was lost by hydrolysis over the following 5 days at 50 °C (Fig. 4b inset). The apparent stability of R5P to hydrolysis over 5 days, even at 50 °C, was unexpected, so we measured the stability of commercial R5P under equivalent pH and temperature conditions; we confirmed that R5P is indeed relatively stable to hydrolysis under mild hydrothermal conditions (Online resource 3).

While pH had little effect, synthesis of R5P was slightly lower at pH 11 (Fig. 4a,b), reflecting the formation of tetra-acetylated ribose at pH 11 alone (Online resource 4). This corresponds to the known p*K*_a_ of the OH groups of ribose, which is ~11.8 (Sen et al. 2014) hence these are acetylated only at quite strongly alkaline pH. The presence of other ions lowered the rate of R5P synthesis and tended to increase its rate of hydrolysis at 50 °C (Online resource 5). Ca^2+^ ions (0.15 M) halved the initial rate of synthesis but subsequently had a limited effect on hydrolysis. In contrast, Mg^2+^ ions barely effected the initial rate of synthesis but tended to promote hydrolysis, with ~40% loss over 48 h (Online resource 5). Again this points to the differing roles of Mg^2+^ and Ca^2+^ in the cell, whereby Mg^2+^ promotes both the formation and reactivity of phosphorylated intermediates. Borate ions have previously been claimed to stabilise ribose (Ricardo et al. 2004) potentially favoring phosphorylation to ribose-5-phosphate (Mellersh and Smith 2010) but had little effect in our hands (Online resource 5).

The phosphorylation of adenosine to AMP by AcP was markedly slower, taking several hours even at 50 °C (Fig. 4e–h), but ultimately achieving a similar yield (relative to adenosine) of ~2%. These differences may reflect steric or chemical hindrance by the nucleobase, or the reaction conditions, which differed due to the limited solubility of adenosine (maximum solubility at 20 °C ~10 mM). Again, the concentration of AMP formed at pH 11 was slightly lower; but in general the pH had little effect on phosphorylation in the case of either R5P or AMP. Nor did the presence or absence of Mg^2+^ or Fe^2+^ ions under anaerobic or aerobic conditions (Online resource 6). Although the rate of reaction was slower in this case compared with R5P, all these phosphorylations took place within minutes to hours, rather than days to weeks as commonly reported (Lohrmann and Orgel 1968; Chung et al. 1971). These relatively rapid reaction rates are more commensurate with cellular biochemistry, as catalysed by enzymes, than with much slower geological processes, and so offer a first step towards the far-from-equilibrium dynamics of living cells.

**Fig. 5:**
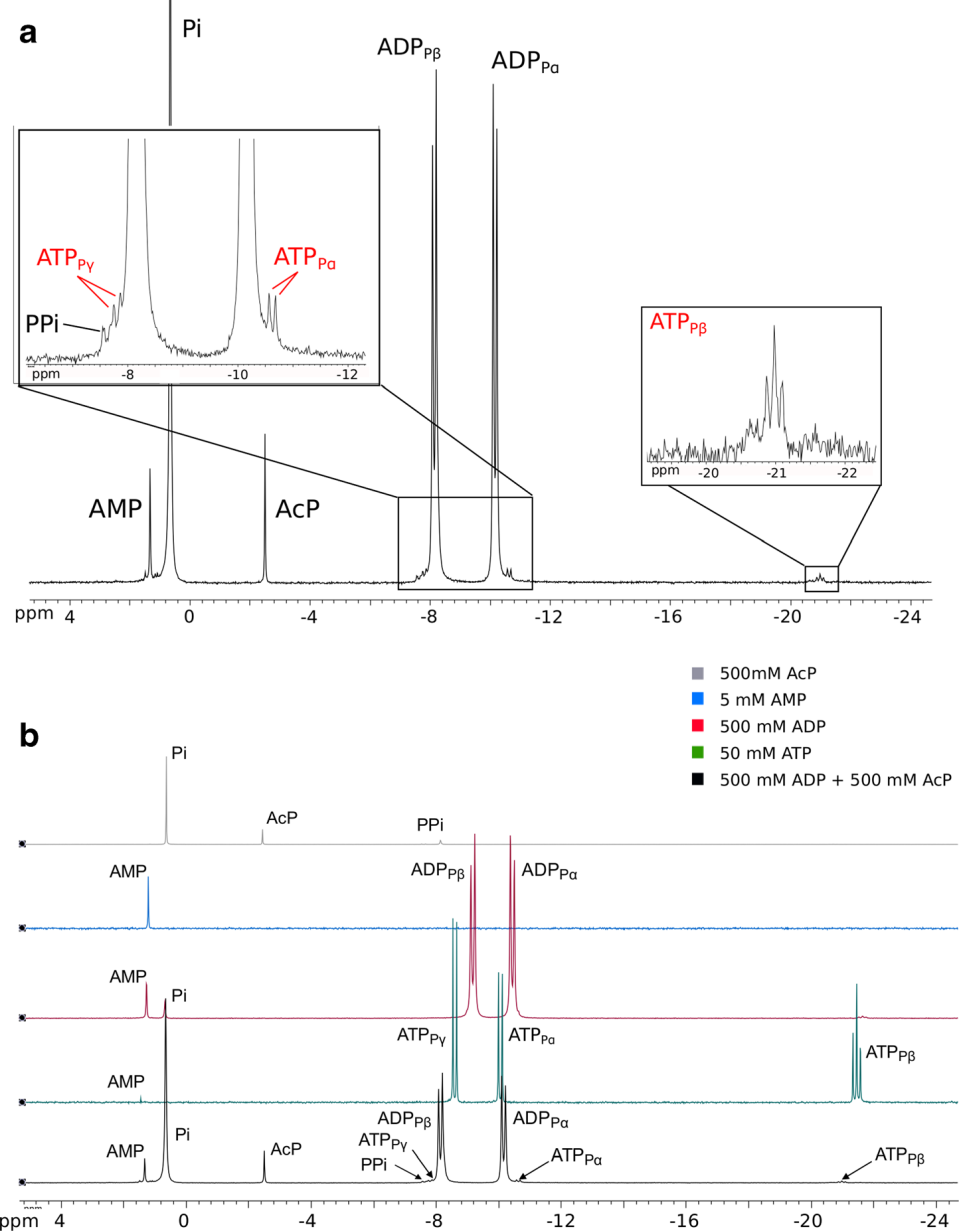
Phosphorylation of ADP by AcP. **a** ^31^P–NMR spectrum of the phosphorylation of ADP by AcP at 50 °C and pH 5.5 after 2 h, yielding ATP. All peaks labelled for clarity. Graph inserts show zoomed in areas of ATP signal. **b** ^31^P–NMR spectra of the phosphorylation of ADP by AcP, compared with commercial ATP, commercial ADP, commercial AMP, and commercial AcP. Small pH differences account for the imperfect alignment of the peaks between −8 and −10 ppm

AcP can also phosphorylate ADP to ATP in water, but we were unable to quantify the ATP yields using ^31^P–NMR, as the concentrations were close to the limits of detection of our instrument (Fig. 5). We could not detect ATP over 24 h at 20 °C, but we did detect ATP synthesis after 1–5 h by both ^31^P–NMR (Fig. 5) and HPLC (Online resource 7) at 50 °C, again suggesting reactivity over time periods of minutes to hours. We plan to develop more sensitive HPLC analyses for ATP synthesis in future, so simply report its successful synthesis here. Our findings corroborate those of Kitani et al. (1995) who reported the synthesis of ATP from ADP using AcP, especially when catalysed by Fe^3+^. Unlike Kitani et al. we found higher yields at higher temperatures.

**Fig. 6:**
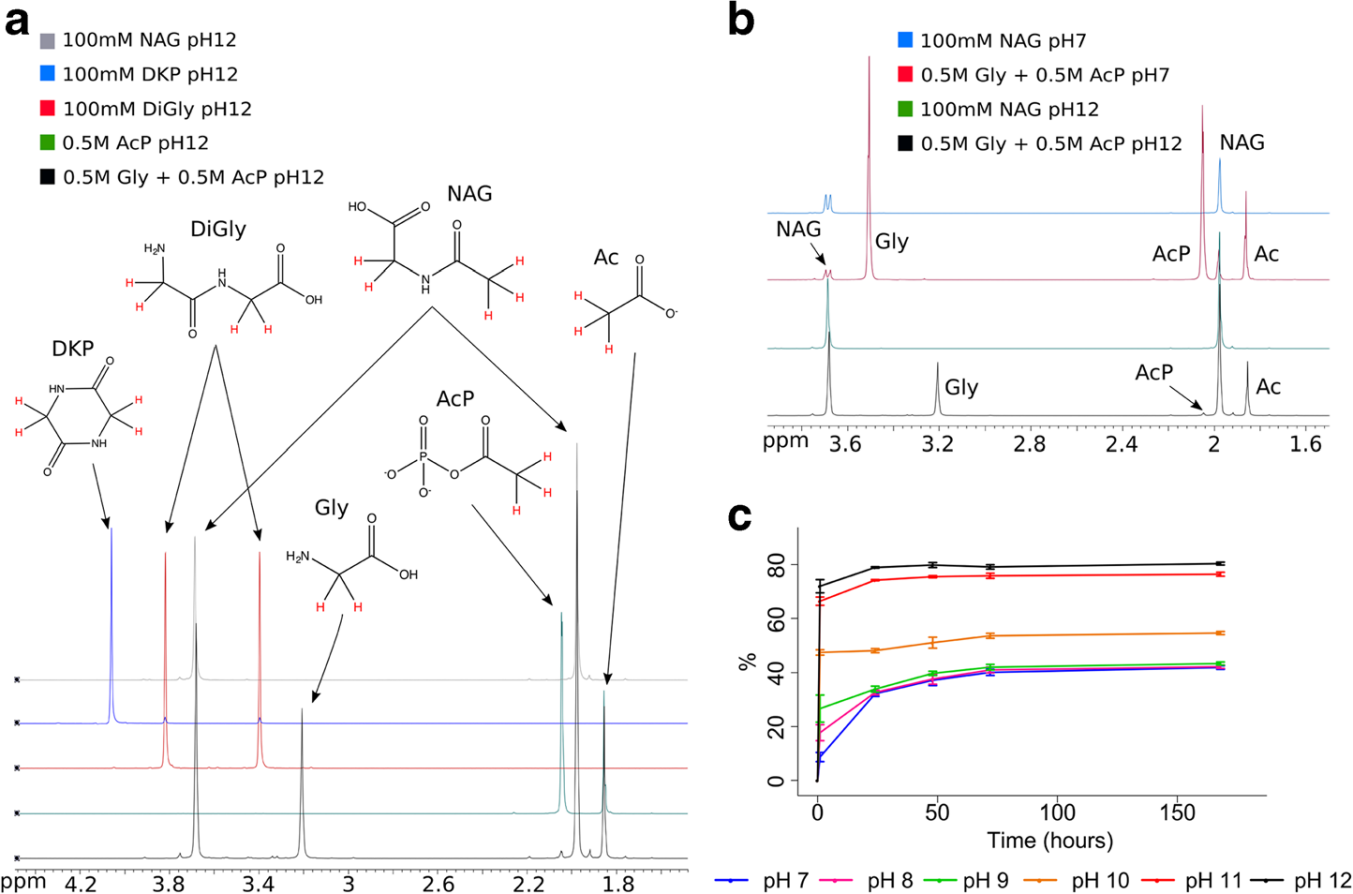
Acetylation of glycine in water by AcP. **a** ^1^H–NMR spectra of acetylation of glycine to N-acetylglycine (NAG) by AcP at 20 °C and pH 12 after 2 h, compared with AcP, diglycine, diketopiperazine (DKP), and NAG; all commercial standards were titrated to pH 12. Molecular structures highlighting the protons giving rise to each spectroscopic peak are added for clarity. **b** ^1^H–NMR spectra of the acetylation of glycine to NAG by AcP at pH 12 and at pH 7 compared with the spectra of commercial NAG at both pH 12 and pH 7. **c** Acetylation of glycine by AcP at 20 °C at different initial pH values over 168 h. N = 3 ± SD

AcP did not polymerise either amino acids or nucleotides in water. Our studies here were limited in that we did not consider polymerization on mineral surfaces or at lower water activity, but only in aqueous solution. Nonetheless, we had hoped to detect short polymers as a first step. We attempted to polymerize glycine in water but were unable to detect any diglycine, diketopiperazine, or any other short polymer; instead we corroborated the findings of others (Di Sabato and Jencks 1961a), that AcP efficiently acetylates the amino group of glycine to form N-acetyl glycine (NAG), especially under alkaline conditions. Diglycine forms two peaks on ^1^H–NMR (Fig. 6) whereas the condensed form of diglycine, diketopiperazine (DKP) forms a single peak. Under alkaline conditions (pH 9–12) we detected two peaks at around 3.7 ppm and 2 ppm (with the exact chemical shift varying slightly with pH) within minutes of adding AcP to glycine in solution. These peaks corresponded to the formation of NAG (Fig. 6a). This interpretation was confirmed by the behavior of NAG by ^1^H–NMR under more acidic conditions (Fig. 6b and Online resource 8). At pH 7 and below, NAG still forms two peaks, but the peak at 3.7 ppm now splits into a doublet. The splitting of the H2-C protons at low pH relates to their interaction with the proton on the neighbouring N. This H-N proton exchanges with protons in the solvent at a rate that depends on pH. At low pH the rate of exchange of the H-N proton is lower than the speed at which that proton affects the H2-C protons (by shielding and un-shielding), so the H2-C signal splits. This pH-dependent peak splitting at 3.7 ppm is diagnostic of NAG and confirms that this was the major product formed, rather than diglycine or DKP. We found an 80% yield (relative to glycine) after 24 h at an initial pH 12 (with ~70% yield after 1 h) falling to <40% yield at an initial pH 7–9 after 24 h (Fig. 6c).

**Fig. 7:**
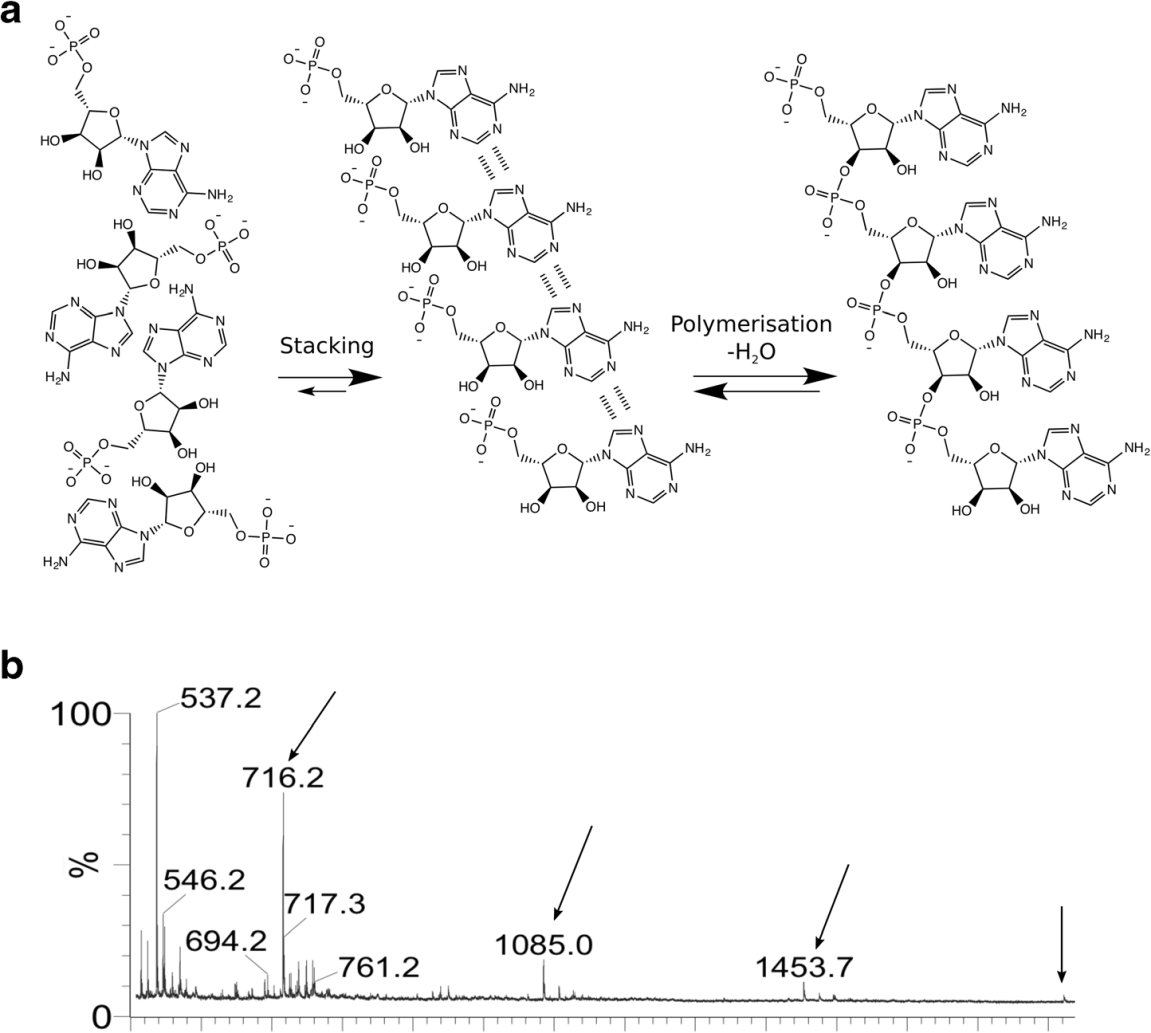
AMP stacking. **a** Diagram showing stacking (middle) and polymerisation (right) of AMP monomers. Stacking is energetically favoured in water due to nucleobase interactions, and even more so when dissolved cations balance the negative charges of AMP. **b** MALDI mass spectrum of the products of the reaction of AMP (3 mM) and AcP (450 mM), at pH 5 and after 24 h of incubation at 20 °C. Other incubation conditions (temperature, pH, time) resulted in similar spectra. 716.2 m/z peak corresponds to 2xAMP (AMP = 347.22 m/z) + Na^+^; 1085.0 m/z peak corresponds to 3xAMP + K^+^ + Li^+^; 1453.7 m/z peak corresponds to 4xAMP + Na^+^ + K^+^ + Li^+^; 1824.1 m/z peak corresponds to 5xAMP + 2xNa^+^ + K^+^ + Li^+^

AcP did not promote the polymerization of AMP in water, but we did detect aggregated stacks of up to 7 monomers, which have been suggested to favour polymerisation, at least of cyclic nucleotides through a base catalysed ‘click-type’ mechanism (Costanzo et al. 2009; Costanzo et al. 2012). We examined the behaviour of non-cyclic AMP, as others have successfully polymerized imidazole-activated AMP in water on clay catalysts (Burcar et al. 2015). Aggregates are easily mistaken for polymers (Burcar et al. 2013), but differ from them in that one water-equivalent is lost for each monomer that is added to a polymer, due to the condensation necessary to generate the new covalent bond (Fig. 7a). Using MALDI-TOF, the ion fragments indicative of AMP polymerisation are therefore: 2-mer (675.44 m/z), 3-mer (1004.66 m/z), 4-mer (1333.88 m/z) and successive additions of 329.22 m/z, corresponding to an AMP residue (AMP (347.22 m/z) – H_2_O (18.01 m/z) incorporated into the growing RNA polymer.

We initially thought that we had produced AMP polymers, as aggregates were only formed upon the addition of AcP. However, the peaks detected were at 716.3, 1085.1, 1453.4, 1821.74, 2190.8, 2558,37 m/z, marked by arrows (Fig. 7b). These do not correspond to AMP polymers, but could reflect the presence of adducts of nucleotide aggregates with counter-ions. We therefore ran experiments with alternative cations K^+^ and Li^+^ replacing Na^+^ as a counter-ion to AcP. Online resource 9 shows that the peak at 1085.1 m/z appeared only when the original AcP solution (which contained a high Na^+^ concentration from the preparation protocol) was added. The peaks detected varied with the most common cation present (generating different aggregates) and not as a result of polymerisation. So the addition of AcP promoted AMP aggregation in our first experiments because the counter-ion, Na^+^, stabilizes the formation of aggregated stacks containing up to 7 AMP monomers. While these findings were disappointing, it remains possible that AcP could promote nucleotide polymerization on mineral surfaces or at lower water activity.

## Discussion

We show that AcP is formed at modest (~2%) yields within minutes under ambient conditions (neutral pH and 20 °C) and mild hydrothermal (pH 8, 50 °C) conditions, from the simple 2-carbon precursor thioacetate (Fig. 2). We did not detect the formation of AcP from the simple thioester methyl thioacetate under any of the conditions tested. While most theoretical work (de Duve 1991; Martin and Russell 2007) has considered methyl thioacetate as a prebiotic equivalent to acetyl CoA, thioacetate has also been synthesised from CO and CH_3_SH under mild hydrothermal conditions (Huber and Wächtershäuser 1997). Being even simpler, thioacetate is arguably a more plausible prebiotic precursor, if not a thioester. Once formed, AcP exhibits an ideal poise between stability and reactivity: it is stable over >5 h under ambient conditions, even at pH 11 and in the presence of Mg^2+^ and Ca^2+^ ions (Fig. 3). Earlier work showed that AcP is hydrolysed much more rapidly above pH 11 (Koshland 1952; Etaix and Buvet 1975), but those conditions would be rare even in alkaline hydrothermal systems (Kelley et al. 2001; 2005) and we did not study them here. AcP is less stable at warmer temperatures: it is completely hydrolysed over 3–5 h at 50 °C, and within 90 min at 60 °C (Fig. 3). AcP does indeed act as an ATP mimetic, phosphorylating several intermediates in nucleotide synthesis, in water, over minutes to hours: ribose to ribose-5-phosphate (Fig. 4), adenosine to AMP (Fig. 4), and ADP to ATP (Fig. 5). But AcP did not promote the polymerization of either amino acids (glycine; Fig. 6) or nucleotides (AMP; Fig. 7) in aqueous solution. Instead, it tended to acetylate the amino group of amino acids (Fig. 6), especially at more alkaline pH, as reported by others (Di Sabato and Jencks 1961a). AcP can also acetylate all four hydroxide groups on ribose at strongly alkaline pH (11), albeit in quite low amounts (Online resource 4). Its tendency to acetylate amino groups could partially explain why AcP is used less commonly than ATP to catalyse phosphorylation reactions in modern cells, and indeed in archaea AcP remains bound to the active site of acetyl CoA synthetase (Bräsen et al. 2008; Schönheit et al. 2016).

While the yields of phosphorylated products were generally low (~2%) we do not consider that to be a problem; rather the reverse. As argued by de Duve (2005), the ‘only scientifically plausible’ explanation for the emergence of biological catalysts, whether ribozymes or enzymes, is selection. The first biological catalysts must have been selected because they enhanced flux through proto-metabolic pathways. These proto-metabolic pathways presumably had much the same substrates and products, and probably many equivalent intermediates, as later genetically encoded pathways (Copley et al. 2007; Martin et al. 2014; Sojo et al. 2016; Keller et al. 2017a). Tardy reactions should have been promoted by mechanisms equivalent to those still observed in modern cells, such as phosphorylation. That is what we did find. AcP is formed under mild prebiotic conditions; it is capable of phosphorylating biologically meaningful molecules, including ADP to ATP; and it is still used by cells today, critically as the fulcrum between thioester and phosphate metabolism (de Duve 1991; Ferry and House 2006; Schönheit et al. 2016). If metabolism did indeed emerge through selection, then low yields of intermediates and products would be expected to form in water in the absence of catalysts. Work on prebiotic forms of glycolysis and the pentose phosphate pathway (Keller et al. 2014), TCA cycle (Keller et al. 2017b) and gluconeogenesis (Messner et al. 2017) suggests that the intermediates do indeed form spontaneously under prebiotic conditions. These intermediates are necessarily relatively reactive, or they would accumulate and could not be part of metabolic networks (Smith and Morowitz 2004; Keller et al. 2017a).

The first steps of catalysis should be promoted by ions or mineral clusters equivalent to the cofactors of modern proteins. Simple ions in solution should improve yields slightly. Accordingly, we found that Mg^2+^ promoted the synthesis of AcP from thioacetate relative to other ions. That is important because Mg^2+^ is unusual in being able to complex with two phosphate groups (Holm 2012); it is still required in cells today to maintain the structure of RNA and enzymes, as well as the activity of phosphorylated molecules, including ATP. Chelation of ions or small mineral crystals (such as FeS clusters) by amino acids or short polypeptides should enhance catalysis further, as these complexes ought to mimic the active site of enzymes for reasons of physical chemistry, increasing yields further (Milner-White and Russell 2005, 2011; Copley et al. 2007; Nitschke et al. 2013; West et al. 2017). Ultimately, genetically encoded ribozymes or enzymes would increase yields considerably more. In other words, the explicit prediction of a selectionist approach to the origin of life is that the yields of prebiotic reactions should be low in the absence of organic catalysts in water. From this perspective, the synthetic chemists’ preoccupation with high prebiotic yields of specific products is misguided.

The fact that AcP did not drive the condensation of amino acids or nucleotides, even to a limited degree, was disappointing, but possibly significant. We had hoped that short polymers would form spontaneously in water in the presence of AcP. In the case of glycine, we generated high yields (up to 80%) of N-acetyl glycine, as reported by others (Di Sabato and Jencks 1961a). NAG did not react any further, although acetylation of amino groups can promote the formation of short peptides in the presence of amino acid esters, AMP, a carbodiimide and 1-ethylimidazole, as acetylation of the amino group facilitates reaction of the carboxylic acid group instead (Jauker et al. 2015; Griesser et al. 2017). But modern cells do not promote polymerization using any of these condensing agents, nor through acetylation or phosphorylation of amino groups, as reported for diamidophosphate (Gibard et al. 2017), or acetylation of hydroxides (Bowler et al. 2013); nor do they do so through wet-dry cycles (Rajamani et al. 2008; DeGuzman et al. 2014; Da Silva et al. 2015; Forsythe et al. 2015). Polymerization of cyclic nucleotides in water at temperatures above 75 °C has been reported (Costanzo et al. 2009, 2012) but these findings have proved ‘erratic’ to repeat (Morasch et al. 2014; Šponer et al. 2015). Polymerization apparently occurs only in the absence of Na^+^ ions, and ideally in a gradually desiccating environment (Šponer et al. 2015) that has little in common with polymerization in modern cells.

It could be that condensation reactions really did begin through radically different mechanisms, which were later overwritten by enzymatic catalysis. If metabolism were ‘invented’ in an RNA world that would arguably need to be the case. On the other hand, if intermediary metabolism arose initially from geochemical flux, via conserved intermediates, then it might be that polymerizations in water are genuinely difficult to achieve, and arose later in a world of organic monomer catalysts. If that were the case, the fact that AcP promoted the synthesis of ATP from ADP – as it still does in modern cells (Ferry and House 2006; Schönheit et al. 2016) – might be important, as both amino acid and nucleotide polymerization depends on nucleotide triphosphates today. The concept of a ‘monomer world’ is not new (Copley et al. 2007), and could be highly structured, as the formation of fatty acids is favoured under a wide range of conditions (Amend et al. 2013), and spontaneously form bilayer vesicles when above a threshold concentration (Morowitz et al. 1988; Segré et al. 2001). The possibility of a rudimentary form of heredity, based on several linked positive feedbacks in growing protocells, is consistent with the idea that polymerization to form macromolecules occurred relatively late (West et al. 2017). If so, then AcP could potentially drive prebiotic chemistry towards a form of proto-metabolism that prefigures the metabolic pathways of modern cells, in which relatively unreactive precursors are activated through phosphorylation. AcP therefore begins to bridge the gap between prebiotic chemistry and monomer biochemistry in living cells, but does not directly promote the formation of macromolecules.

The phosphorylation reactions reported here required high concentrations of reagents, and could be criticised for being prebiotically implausible (McCollom 2013). While the concentrations we used are similar to those reported for most other studies (Lohrmann and Orgel 1968; Rabinowitz 1970; Chung et al. 1971; Yamagata et al. 1991; Bujdák and Rode 1996; Liu and Orgel 1997; Huber et al. 2003; Leman et al. 2004; Costanzo et al. 2007; DeGuzman et al. 2014; Morasch et al. 2014; Burcar et al. 2015), three factors make these high concentrations more realistic in alkaline hydrothermal systems. First, deep-sea hydrothermal systems are at high pressure (100–300 Bars) which increases the solubility, and so concentration, of gases such as H_2_. Pressure also increases the likelihood of molecular collisions, which is equivalent to increasing the concentration of reactants or raising the temperature. Continuous hydrothermal flux sustains the critical disequilibria in H_2_, CO_2_ and proton concentrations, and should theoretically drive organic synthesis, including formation of thioacetate and acetyl phosphate, under these conditions (Amend and Shock 1998; Shock and Canovas 2010; Amend et al. 2013; Sojo et al. 2016). Second, thermal gradients and convection currents in alkaline hydrothermal vents can concentrate organics by thermophoresis. Steep heat gradients produced by lasers in closed glass capillaries concentrate nucleotides and small RNAs by 10^3^–10^12^-fold (Braun and Libchaber 2002; Baaske et al. 2007; Mast and Braun 2010; Mast et al. 2013). In simulated hydrothermal systems, we have shown that thermophoresis across an open, inert microporous matrix can concentrate small organics equivalent to nucleotides by at least 5000-fold (Herschy et al. 2014). Third, the charge on phosphorylated biomolecules such as acetyl phosphate makes them more likely to interact with mineral surfaces within hydrothermal vents, promoting their retention in vent pores rather than their discharge from the vent along with uncharged ‘waste’ molecules such as methane (Westheimer 1987; Pratt 2006; Mellersh and Smith 2010). Similarly, if monomers were formed inside fatty acid vesicles, with ‘autotrophic’ growth driven by geologically sustained gradients across the membranes (West et al. 2017), molecular crowding and membrane surface associations inside growing protocells would also increase local concentrations. These three factors mean that the concentrations used in this study, while high, are not necessarily unreasonable.

In conclusion, we report that AcP is readily synthesised under ambient and mild hydrothermal conditions, and promotes phosphorylation reactions in water, without catalysts, under a wide range of conditions. Most of the conclusions of this paper apply equally to other prebiotic environments, and do not need to be interpreted in a hydrothermal context. All reactions reported here, including those under ambient conditions, occur over time scales of minutes to hours, giving dynamics that approach those of living cells, rather than geological systems, even in the absence of catalysts. We believe that these findings make AcP a credible primordial energy currency, coupling carbon and energy flux at the origin of life. The fact that AcP promotes phosphorylation but not condensation reactions in water points to a period of monomer biochemistry before the emergence of polymeric enzymes or ribozymes at the origin of life (Copley et al. 2007). This conception is consistent with autotrophic origins and rudimentary heredity in growing protocells before the emergence of RNA, polypeptides, and true genetic heredity (West et al. 2017). The biochemical utility of AcP may be limited by its tendency to acetylate amino groups. But in driving the synthesis of ATP from ADP, AcP might have helped bridge the gap between monomer biochemistry and the origin of genetic replicators.

## Methods

### Synthesis of Acetyl Phosphate from Thioacetate

Solutions of inorganic phosphate (Na_2_HPO_4_, 20 mM) in 10% D_2_O and Milli-Q water (18 M**Ω**) were prepared with different concentrations (0, 2, 10, 20 mM) of either calcium or magnesium ions (MgCl_2_, CaCl_2_) or an equimolar mix of both. Thioacetic acid (CH_3_COSH, 40 mM) was then added and the pH adjusted to 6, 7 or 8 with aqueous HCl or NaOH (1 M). Samples (600 μL) were taken at time points (0, 10, 20, 30, 40, 50, 60, 120 and 180 min) and immediately frozen at −80 °C. Experiments were carried out at 20 °C and 50 °C. Samples were defrosted and analysed using ^1^H–NMR (Bruker Avance 600 MHz; 10% D_2_O, 16 scans, water suppression) with potassium hydrogen phthalate (KHP, 1 mM) as an internal standard. Concentrations were calculated using the integrated ratio of protons in the internal standard compared to AcP. Standards of AcP were measured with ^1^H–NMR to confirm the shift of the detected peak within the experiments. KHP was detected between 7.4–7.6 ppm and AcP at 2.1 ppm. All peaks in NMR were identified by analysis of both pure standards and spiking of experimental samples. To every time point 15 min was added to take into account time taken from defrosting to start of analysis.

For the results in the supplementary materials, the same experiments were carried out at pH 11 with calcium and magnesium ions, at pH 7 with Fe^2+^ ions at differing concentrations (200, 400 and 1000 μM) in anaerobic conditions, and at pH 7 in both aerobic and anaerobic conditions.

### Phosphorylation of Ribose in Water

Solutions of D-ribose (0.3 M) and AcP (0.3 M) were prepared and, when applicable, aqueous solutions containing MgCl_2_, CaCl_2_ or boric acid (H_3_BO_3_) were added (0.15 M). Experiments were performed at pH 7, 9 and 11 at 20 °C and 50 °C, and samples taken at 2, 30, 60, 120, 300 min, and 1, 2 and 5 days. Quantification was performed using LC-MS (Finnigan LTQ Thermo Fischer; Accela 600 pump and autosampler; column: Hypersil Gold (150 × 2.1 mm, 1.9 μL), in ESI positive mode. Mobile phase solvents were (A) water/0.1% formic acid (FA) and (B) acetonitrile/0.1% FA, at a flow rate of 200 μL/min. The optimal solvent gradient started with 98% A, and went gradually down to 5% A at 18 min; after holding 5% A for 1 min, the gradient went to the initial conditions (98% A) for the last 5.5 min. Samples were derivatised using 3-amino-9-ethylcarbazole (Han et al. 2013) and further purified using solid phase extraction (SPE) protocol (HyperSep, C18 500 mg/mL), followed by a 0.22 μm microfiltration. Ribose-5-phosphate was identified using retention time, and primary and MSMS fragmentation pattern of standards; quantification was performed using calibration curves for each experimental condition (pH and ion concentration). Electron ionisation mass spectrometry showed the precursor ion 425.14 m/z for the derivatised ribose-5-phosphate; this was used for quantification. Molecular ion 521.13 m/z was also monitored, which corresponds to tetraacetylated ribose at pH 11.

### Phosphorylation of Adenosine in Water

Solutions of adenosine (2 mM) and AcP (800 mM) were prepared and pH adjusted to 7, 9 or 11. Samples (500 μL) were taken at 1, 5, 24, 48 and 144 h, diluted to 1 mL with Milli-Q water (18 M**Ω**) and frozen. Samples were analysed on a Dionex HPLC, using a TELOS AT dC18 (100 × 4.6 mm) column, with mobile phases; A: 150 mM KH_2_PO_4_ and 150 mM KCl, B: 15% *v*/v acetonitrile in phase A. Peaks were identified using pure standards and spiked samples and quantified using standard calibration curves.

### Phosphorylation of ADP in Water

Equimolar solutions (0.5 M) of ADP and AcP were prepared and mixed. The pH was adjusted to 5.5 with aqueous HCl or NaOH (1 M) and samples (500 μL) were taken at time-points (1, 2, 3, 4 and 5 h) and immediately frozen at −80 °C. Experiments were carried out at 20 and 50 °C. Samples were thawed and analysed using ^31^P–NMR (^1^H decoupling, Bruker Avance 400 MHz, 152 scans) and peaks were identified using pure standards. The quantification and statistical analysis were carried out by extrapolating the absolute intensity of the peaks of interest (in the case of ATP, the average absolute intensity of the three peaks formed by the β-phosphate, P_β_) and using standard calibration curves.

For the results in the supplementary materials, the same experiments were carried out with mixed equimolar solutions (250 μM) of ADP and AcP at pH 5.5 at 50 °C. Samples (1 mL) were taken at time-points (1, 2, 3, 4 and 5 h) and frozen at −80 °C. Samples were thawed and analysed on a Dionex HPLC, using an Acclaim 120 C18 2.2 μm column (2.1 × 250 mm), with mobile phases A: 0.1 M ammonium acetate, B: 100% acetonitrile. The column flow rate was set at 0.1 mL/min, and the column temperature was maintained at room temperature. The optimal elution gradient was determined to be: from 100% to 95% A during the first 5 min, then up to 75% A for 6.3 min and held at 75% A for 8.7 min. Peaks were identified using pure standards and spiked samples and quantified using standard calibration curves.

### Acetylation of Glycine

Solutions of glycine (0.5 M) were prepared, pH adjusted to 7, 8, 9, 10, 11 or 12 using NaOH (1 M), and AcP (0.5 M; prepared as in Crans and Whitesides 1983), or using a commercial preparation) added to each pH condition. Samples were incubated at 20 °C for 7 days. Analysis was carried out by ^1^H–NMR (Bruker Avance 300 MHz; 10% D_2_O, 16 scans, water suppression). All peaks in NMR were identified by analysis of both pure standards and by spiked experimental samples. Percent conversion of glycine to N-acetylglycine was calculated using the integrated ratio of protons by comparable integration of peak areas.

### Stacking of Adenosine Monophosphate

Reactions were carried out in 1.5 mL Eppendorf tubes with a final volume of 1 mL containing AMP (3 mM) and AcP (450 mM). No additional ions were added during the initial tests. The tubes were heated when applicable in a block heater to 20 °C, 50 °C or 75 °C, as it has been claimed some condensation reactions only take place at 75 °C and above (Šponer et al. 2015). The pH was adjusted to 5 or 11 using solutions of 5 M HCl and 10 M NaOH. The incubation time for each experiment was of a maximum of 5 h (as little effect of AcP would be expected any longer, especially at high temperature) although some samples were also taken after 24 and 48 h. Samples were not frozen (in order to store them) as the freeze-thaw process could account for some polymerisation, which would be an artefact in these experiments.

A MALDI matrix consisting of 2,4,6-trihydroxyacetophenone monohydrate (THAP) plus ammonium citrate was used. Two solutions were prepared for the MALDI matrix: 0.0372 g of THAP were added to 1 mL of acetonitrile (ACN); 0.045 g of ammonium citrate dibasic were added to 1 mL of HPLC water. Each solution was maintained at 4 °C for a maximum of a week. The required equal volumes of each solution were mixed to obtain the MALDI matrix. A volume of 1 μL of freshly prepared matrix solution was mixed with 1 μL of sample and deposited onto a clean steel MALDI-TOF plate. The liquid volume was allowed to evaporate for 30 min before the introduction of the steel plate into the MALDI-TOF instrument. The MALDI-TOF MS analysis was performed using a Waters micro MX mass spectrometer. The analytical conditions were: reflectron and negative ion mode, 280 au of laser power, 2000 V of pulse, 2500 V of the detector, 12,000 V of flight tube, 5200 V of reflector, 3738 V of negative anode, and 500–5000 amu of scan range. The mass spectrometer was routinely calibrated using a low-molecular-weight oligonucleotide standard (comprising of a DNA 4-mer, 5-mer, 7-mer, 9-mer, and 11-mer (Bruker Daltonics)) as external calibrants. Each oligonucleotide standard was initially dissolved in 100 μL HPLC water, divided in aliquots and frozen at −80 °C. A fresh aliquot was used at each analytical calibration.

## Electronic Supplementary Material

Online resource 1 Synthesis of AcP from thioacetate and orthophosphate at pH 11 at 20 °C. (a) AcP synthesis in the presence of Ca^2+^ and Mg^2+^ ions; 0, 2, 10 or 20 mM of Ca^2+^ plus equimolar concentrations of Mg^2+^, (*N* = 3 ± SD). (b) Mg^2+^ ions alone; 0, 2, 10 or 20 mM, (*N* = 2 ± SD). (GIF 154 kb)

High resolution image (TIFF 687 kb)

Online resource 2 Synthesis of AcP from orthophosphate and thioacetate at pH 7 and 20 °C. (a) Anaerobic experiments carried out in the presence of different concentrations of Fe^2+^ ions (0, 200, 400 and 1000 μM). In the absence of thioacetic acid no AcP was produced; and (b) under anaerobic and aerobic conditions with no ions present. N = 3 ± SD. (GIF 77 kb)

High resolution image (TIFF 502 kb)

Online resource 3 Degradation profile for commercial ribose 5-phosphate over 5 days at pH 7, 9 and 11, stored at (a) 20 °C and (b) 50 °C. N = 3 ± SD. (GIF 207 kb)

High resolution image (TIFF 711 kb)

Online resource 4 Synthesis of tetraacetylated ribose (521.13 m/z) from D-ribose and AcP. (a) 20 °C (b) 50 °C. Acetylation of D-ribose was only detected at pH 11. N = 3 ± SD. (GIF 222 kb)

High resolution image (TIFF 51309 kb)

Online resource 5 Synthesis of ribose 5-phosphate from D-ribose and AcP at pH 9 and 50 °C in the presence of different ions (0.15 M). An equivalent run without ions (black) is shown for comparison purposes. Graph insert show full reaction profile over 120 h. N = 3 ± SD. (GIF 154 kb)

High resolution image (TIFF 675 kb)

Online resource 6 Phosphorylation of adenosine to adenosine monophosphate (AMP) by AcP under anaerobic conditions with the addition of metal ions (Fe^2+^ and Mg^2+^). Experiments at pH 7 and 20 °C. (a) Anaerobic conditions with Fe^2+^ at 200 μM and Mg^2+^ ions at 2 mM. (b) Aerobic conditions with Mg^2+^ ions at 2, 20, 100 and 200 mM. In both experiments phosphorylations did not occur in the absence of AcP. N = 3 ± SD. (GIF 39 kb)

High resolution image (TIFF 294 kb)

Online resource 7 HPLC-UV chromatograms of (a) commercial ATP, ADP and AMP, and (b) experimental phosphorylation of ADP by AcP. (GIF 18 kb)

High resolution image (TIFF 477 kb)

Online resource 8 ^1^H–NMR spectra of commercial N-acetylglycine (100 mM) at pH 2, 5, 7, 9, 10, and 11. In alkaline pH (9–11), the peak at ~3.8 ppm is a singlet, whereas in acidic pH (2–7) it splits into a doublet (see text). (GIF 23 kb)

High resolution image (TIFF 748 kb)

Online resource 9 MALDI-TOF mass spectra of solutions of 3 mM AMP with added (a) 3 mM LiCl, (b) 3 mM KOH, (c) 3 mM NaOH and (d) water. Main peaks corresponding to stacks of AMP molecules with each cation are shown for clarity. The size of an AMP monomer is of 347.22 m/z. (GIF 47 kb)

High resolution image (TIFF 2409 kb)
