# Supplementary figures and images for: Acetyl Phosphate as a Primordial Energy Currency at the Origin of Life
Source: Orig Life Evol Biosph. 2018 Mar 3;48(2):159–79. doi: 10.1007/s11084-018-9555-8 (PMC6061221; doi:10.1007/s11084-018-9555-8)

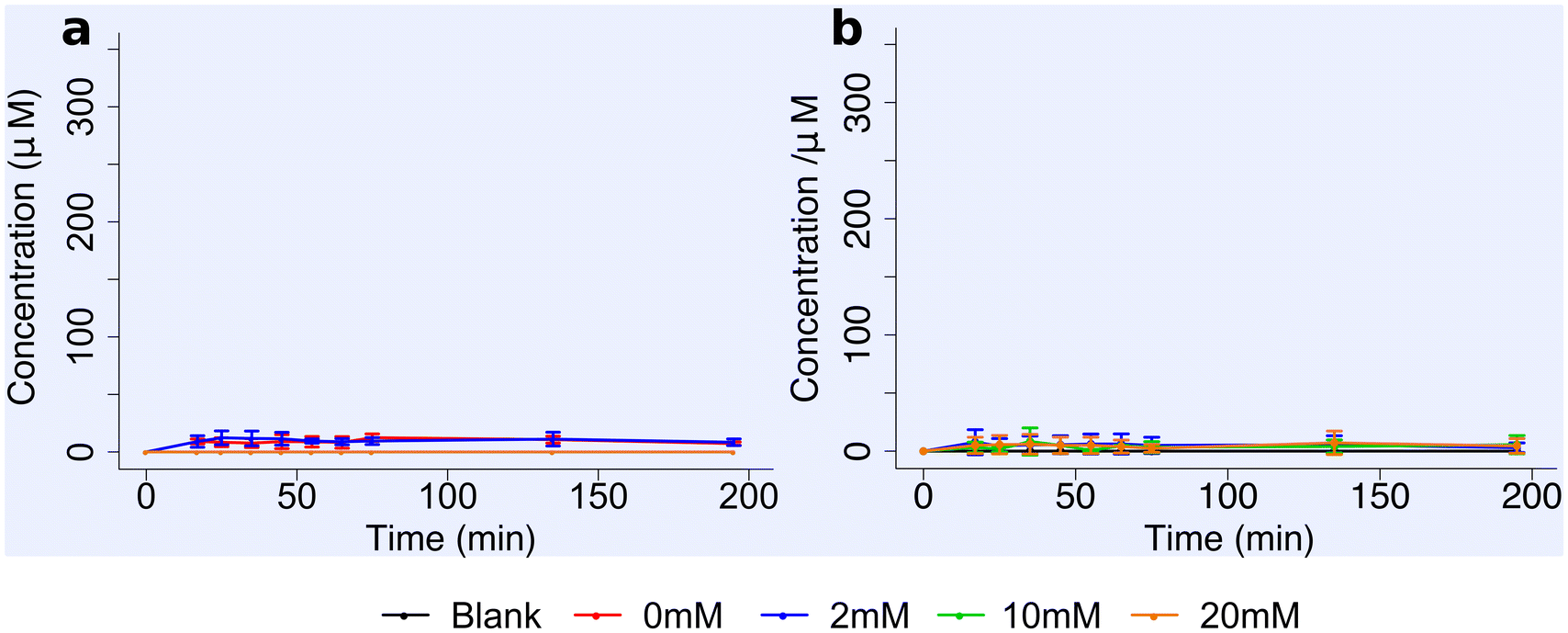

Supplement: Supplementary file 1 — Synthesis of AcP from thioacetate and orthophosphate at pH 11 at 20 °C. (a) AcP synthesis in the presence of Ca2+ and Mg2+ ions; 0, 2, 10 or 20 mM of Ca2+ plus equimolar concentrations of Mg2+, (N = 3 ± SD). (b) Mg2+ ions alone; 0, 2, 10 or 20 mM, (N = 2 ± SD). (GIF 154 kb) [file 11084_2018_9555_Fig8_ESM.gif]

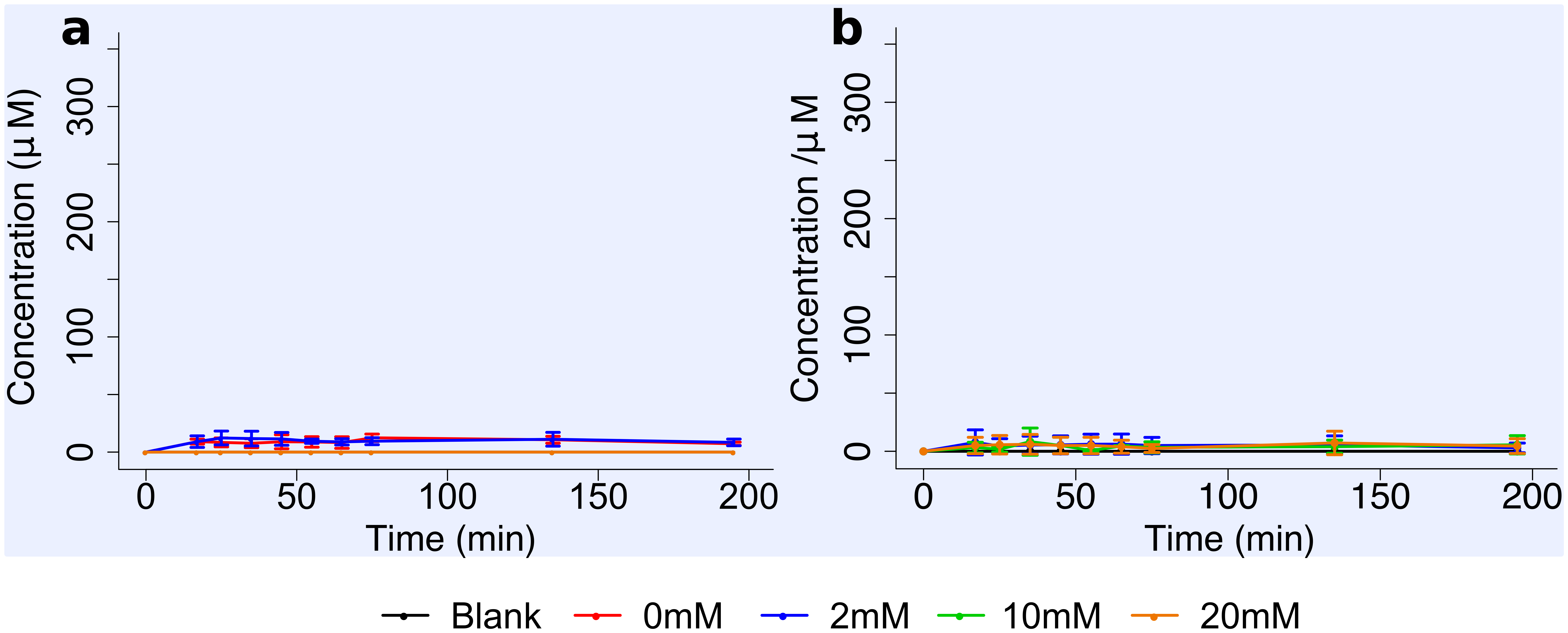

Supplement: Supplementary file 2 — High resolution image (TIFF 687 kb) [file 11084_2018_9555_MOESM1_ESM.tif]

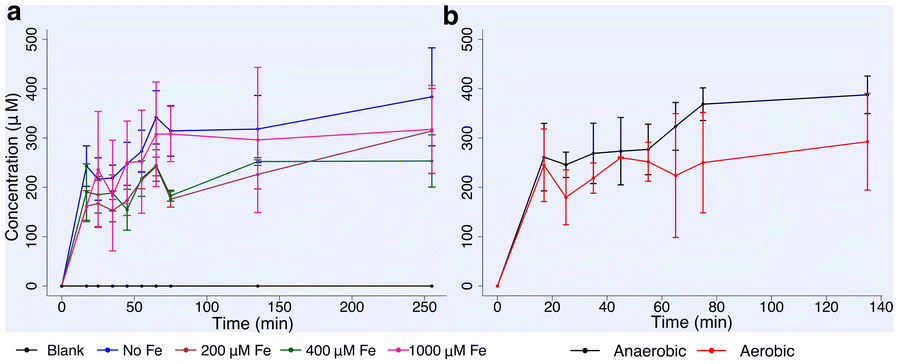

Supplement: Supplementary file 3 — Synthesis of AcP from orthophosphate and thioacetate at pH 7 and 20 °C. (a) Anaerobic experiments carried out in the presence of different concentrations of Fe2+ ions (0, 200, 400 and 1000 μM). In the absence of thioacetic acid no AcP was produced; and (b) under anaerobic and aerobic conditions with no ions present. N = 3 ± SD. (GIF 77 kb) [file 11084_2018_9555_Fig9_ESM.gif]

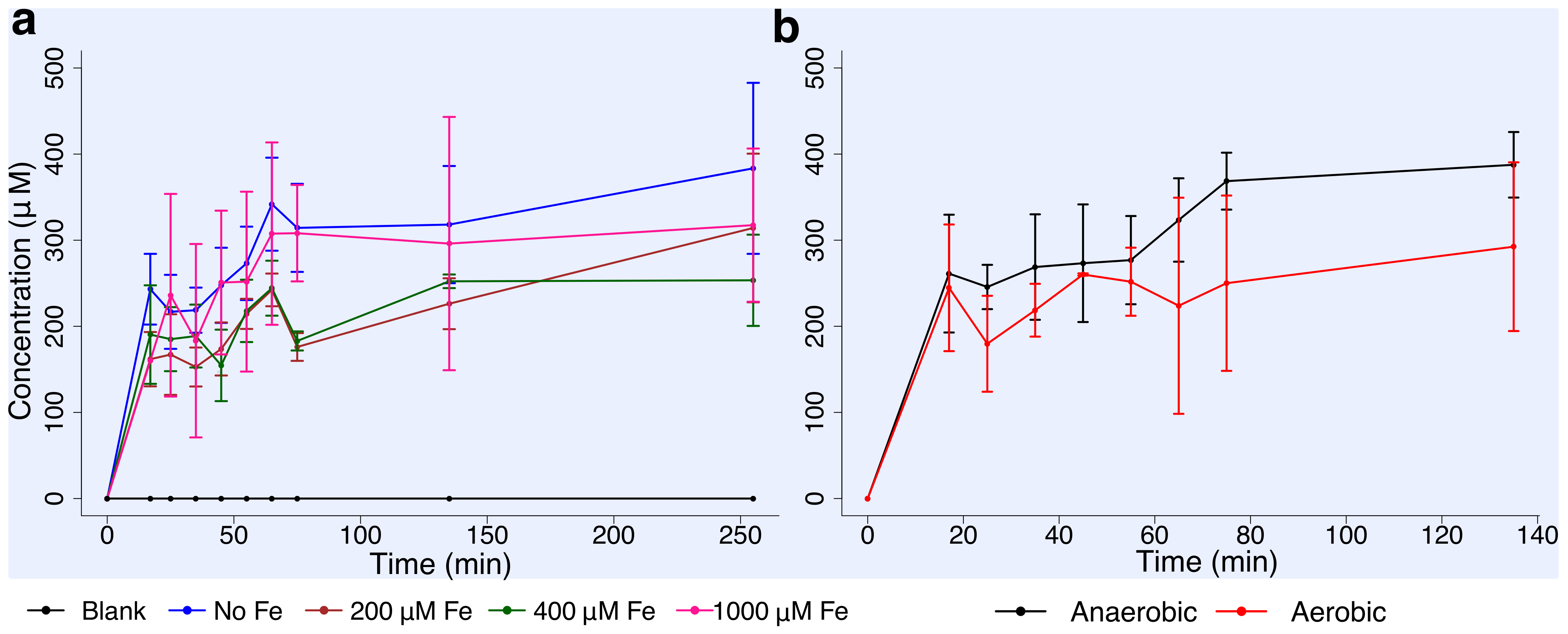

Supplement: Supplementary file 4 — High resolution image (TIFF 502 kb) [file 11084_2018_9555_MOESM2_ESM.tif]

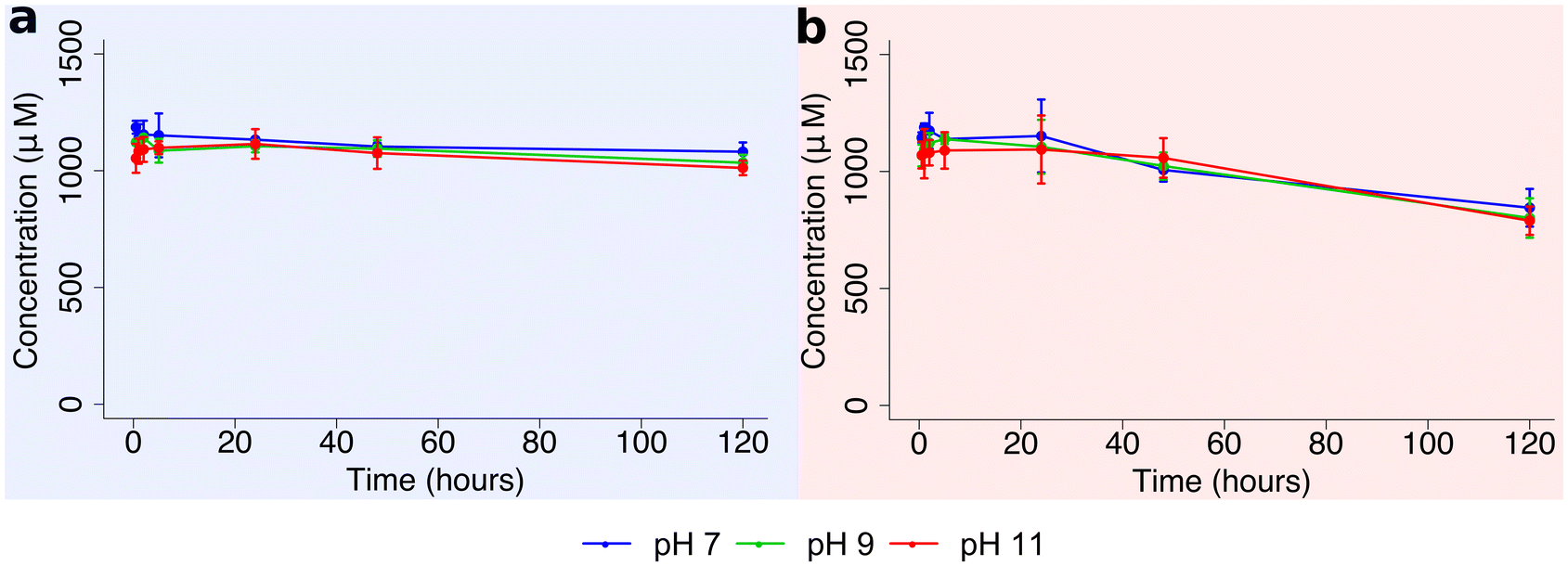

Supplement: Supplementary file 5 — Degradation profile for commercial ribose 5-phosphate over 5 days at pH 7, 9 and 11, stored at (a) 20 °C and (b) 50 °C. N = 3 ± SD. (GIF 207 kb) [file 11084_2018_9555_Fig10_ESM.gif]

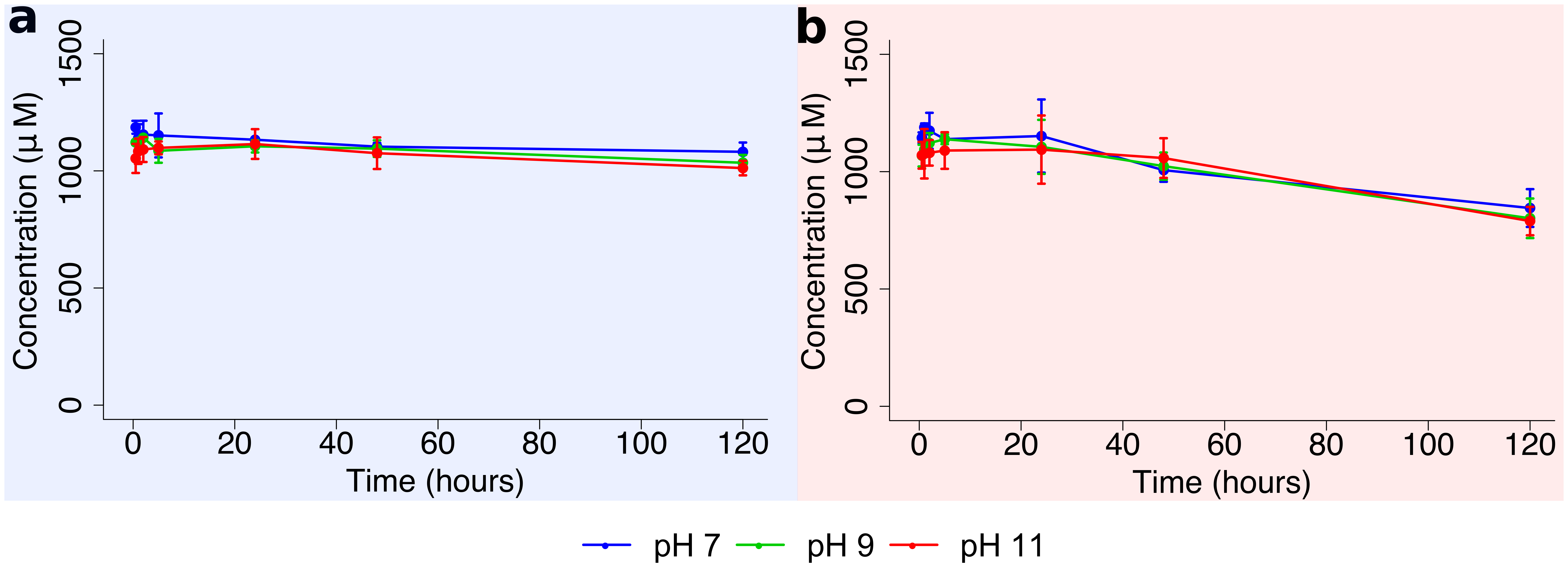

Supplement: Supplementary file 6 — High resolution image (TIFF 711 kb) [file 11084_2018_9555_MOESM3_ESM.tif]

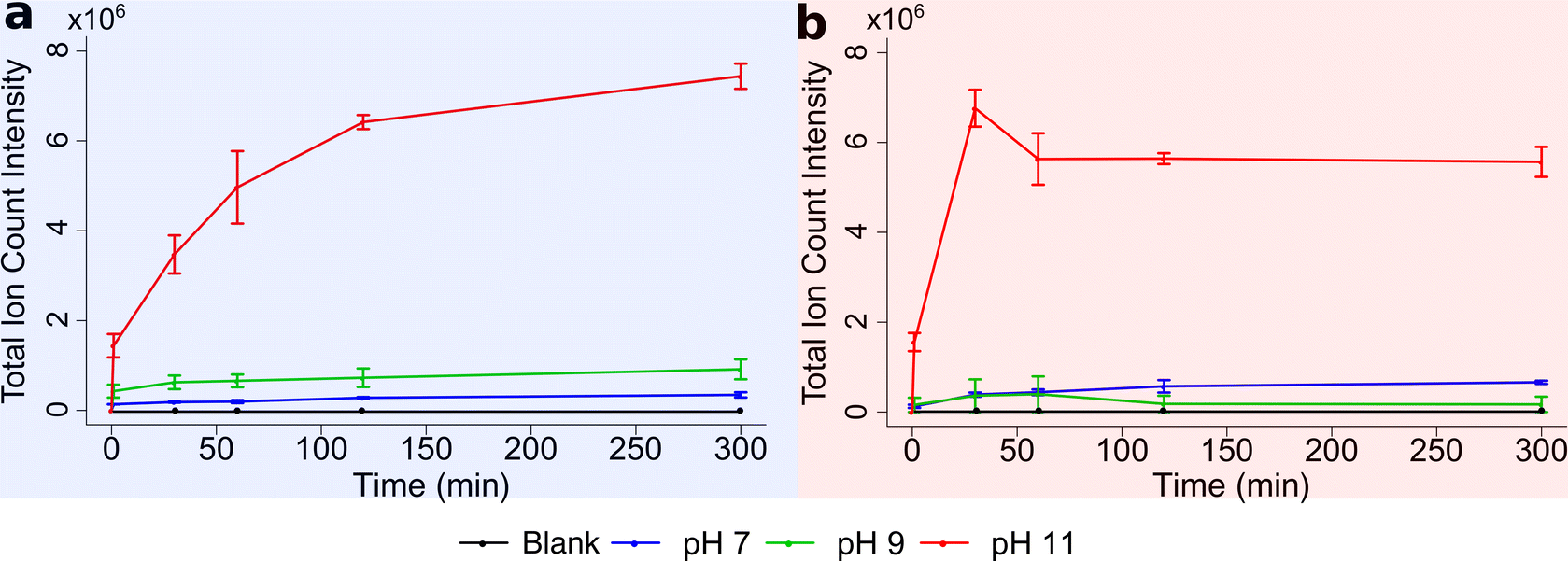

Supplement: Supplementary file 7 — Synthesis of tetraacetylated ribose (521.13 m/z) from D-ribose and AcP. (a) 20 °C (b) 50 °C. Acetylation of D-ribose was only detected at pH 11. N = 3 ± SD. (GIF 222 kb) [file 11084_2018_9555_Fig11_ESM.gif]

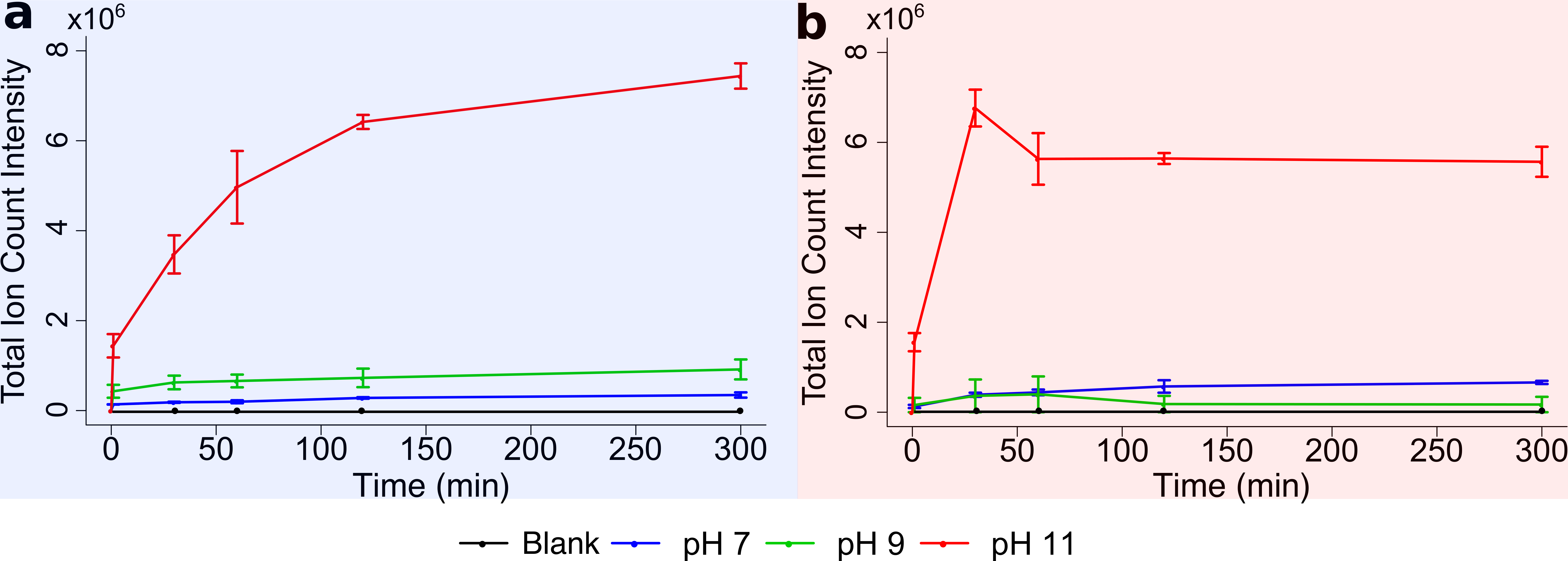

Supplement: Supplementary file 8 — High resolution image (TIFF 51309 kb) [file 11084_2018_9555_MOESM4_ESM.tif]

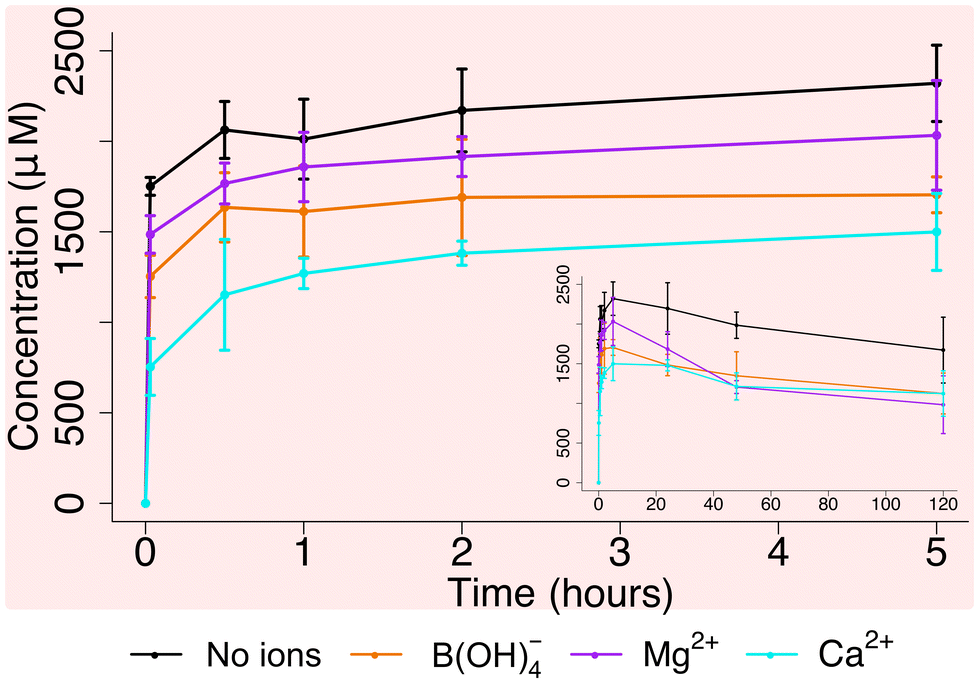

Supplement: Supplementary file 9 — Synthesis of ribose 5-phosphate from D-ribose and AcP at pH 9 and 50 °C in the presence of different ions (0.15 M). An equivalent run without ions (black) is shown for comparison purposes. Graph insert show full reaction profile over 120 h. N = 3 ± SD. (GIF 154 kb) [file 11084_2018_9555_Fig12_ESM.gif]

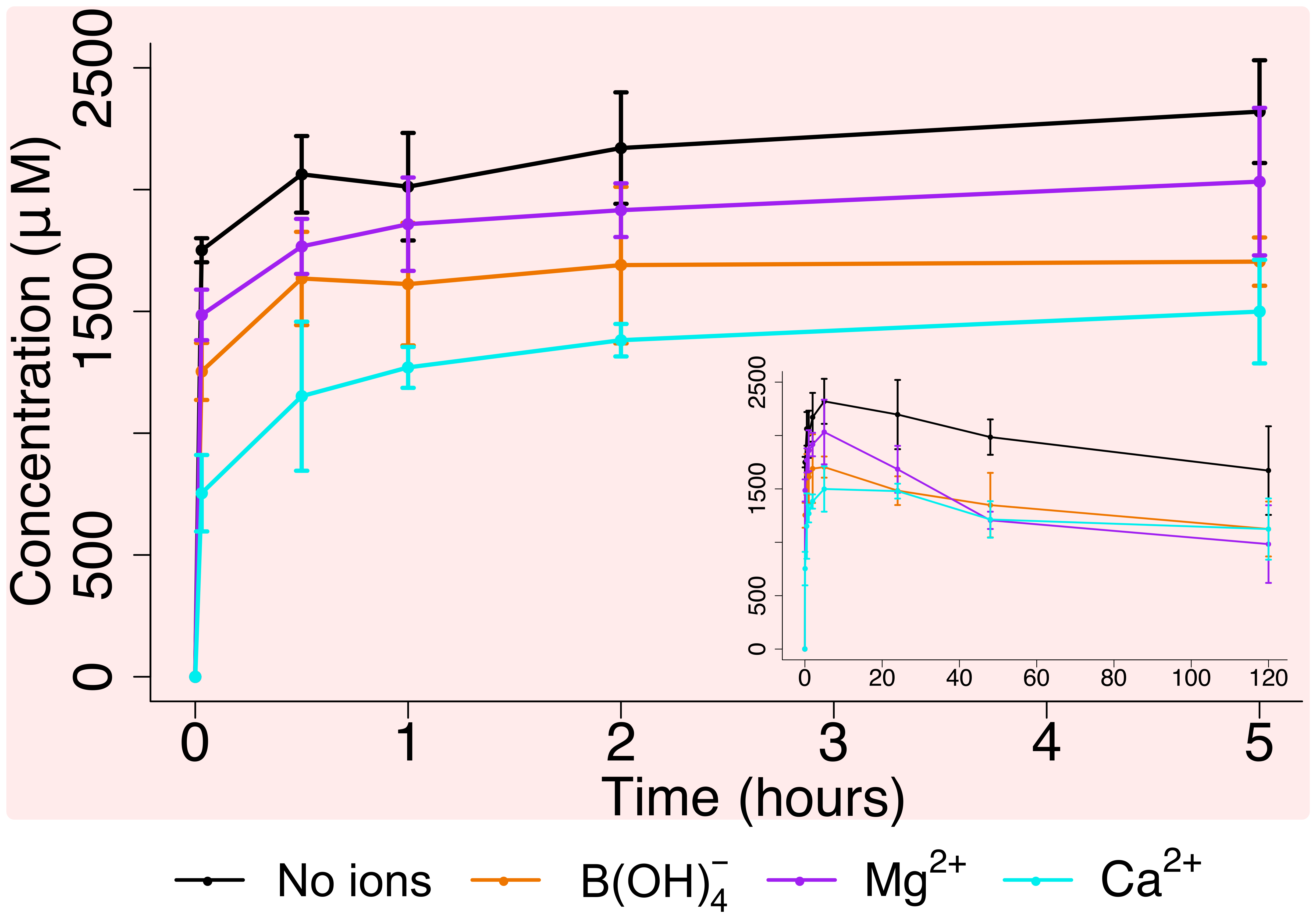

Supplement: Supplementary file 10 — High resolution image (TIFF 675 kb) [file 11084_2018_9555_MOESM5_ESM.tif]

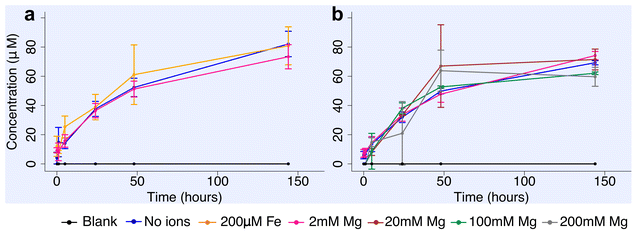

Supplement: Supplementary file 11 — Phosphorylation of adenosine to adenosine monophosphate (AMP) by AcP under anaerobic conditions with the addition of metal ions (Fe2+ and Mg2+). Experiments at pH 7 and 20 °C. (a) Anaerobic conditions with Fe2+ at 200 μM and Mg2+ ions at 2 mM. (b) Aerobic conditions with Mg2+ ions at 2, 20, 100 and 200 mM. In both experiments phosphorylations did not occur in the absence of AcP. N = 3 ± SD. (GIF 39 kb) [file 11084_2018_9555_Fig13_ESM.gif]

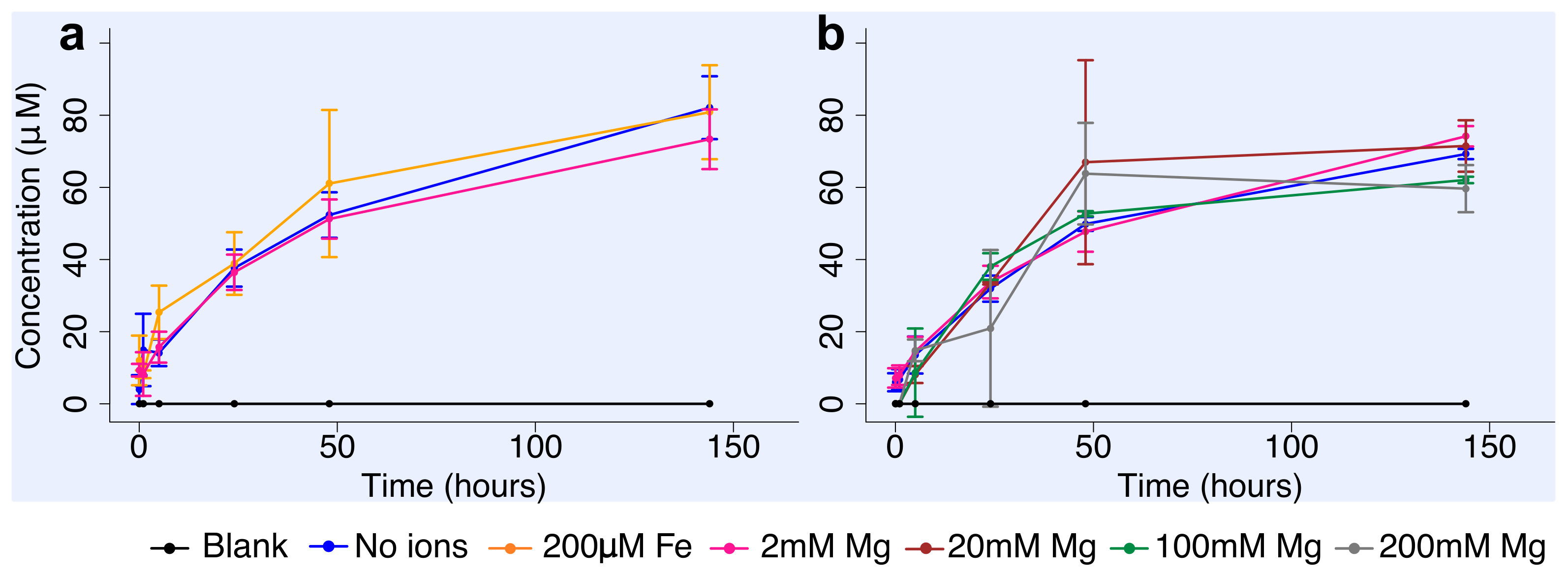

Supplement: Supplementary file 12 — High resolution image (TIFF 294 kb) [file 11084_2018_9555_MOESM6_ESM.tif]

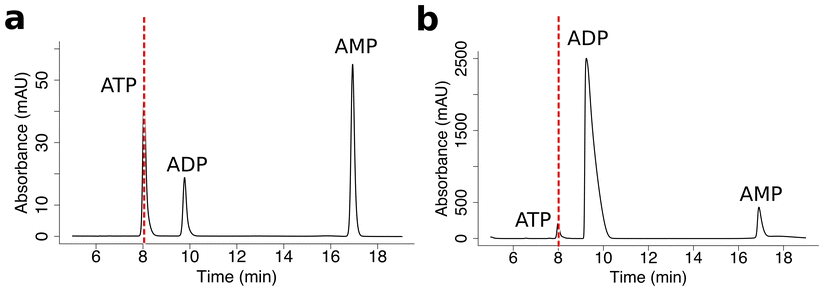

Supplement: Supplementary file 13 — HPLC-UV chromatograms of (a) commercial ATP, ADP and AMP, and (b) experimental phosphorylation of ADP by AcP. (GIF 18 kb) [file 11084_2018_9555_Fig14_ESM.gif]

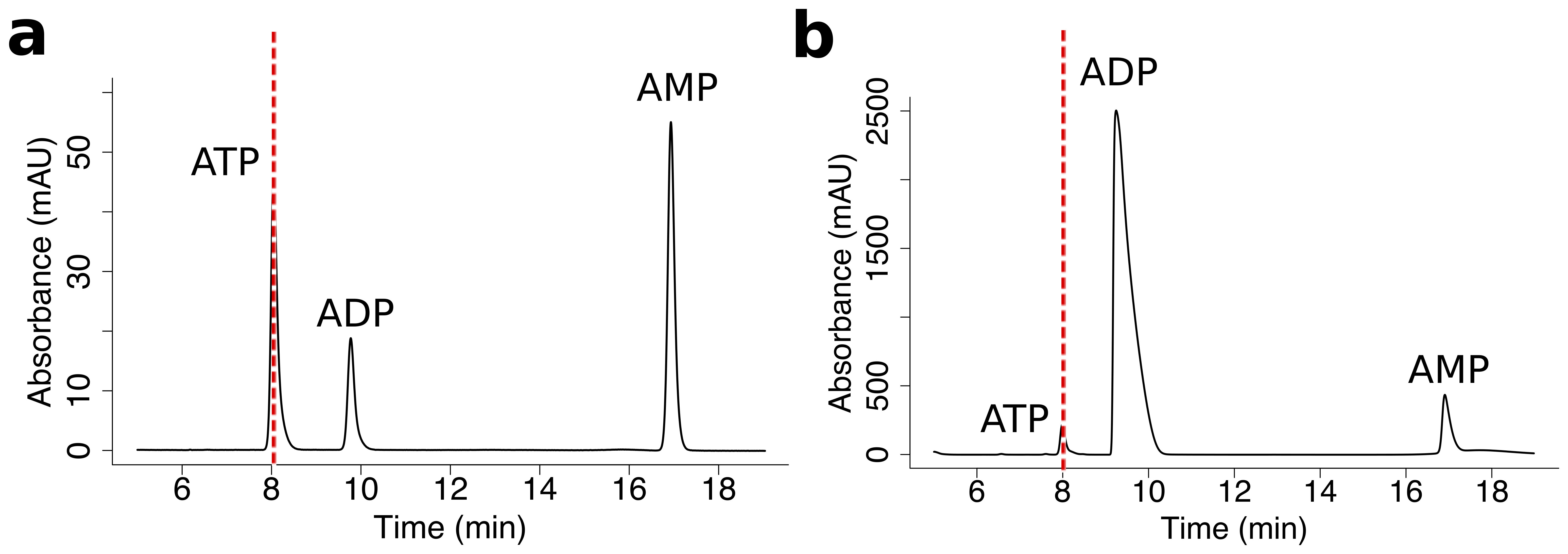

Supplement: Supplementary file 14 — High resolution image (TIFF 477 kb) [file 11084_2018_9555_MOESM7_ESM.tif]

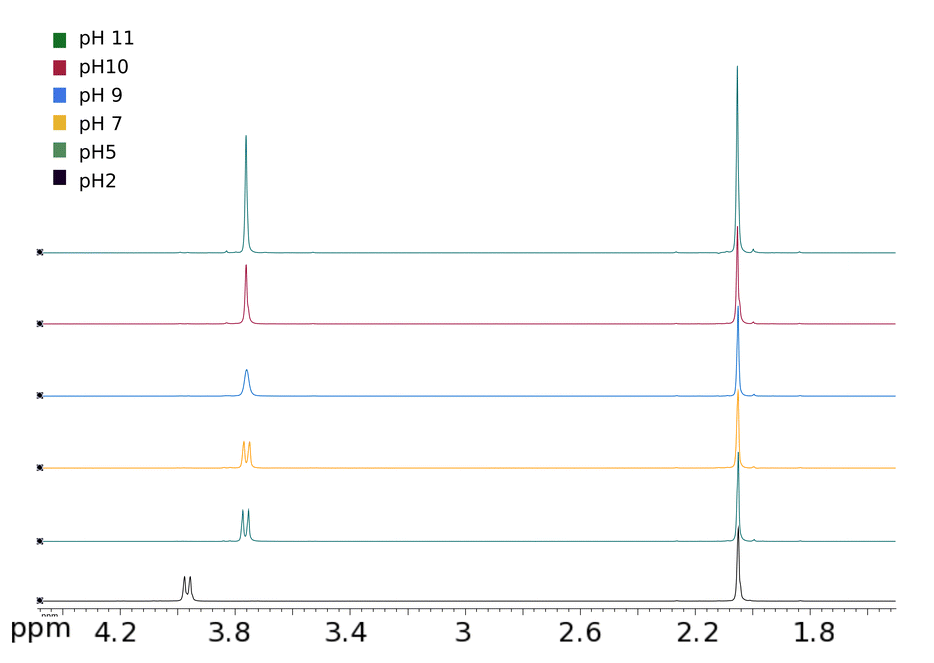

Supplement: Supplementary file 15 — 1H–NMR spectra of commercial N-acetylglycine (100 mM) at pH 2, 5, 7, 9, 10, and 11. In alkaline pH (9–11), the peak at ~3.8 ppm is a singlet, whereas in acidic pH (2–7) it splits into a doublet (see text). (GIF 23 kb) [file 11084_2018_9555_Fig15_ESM.gif]

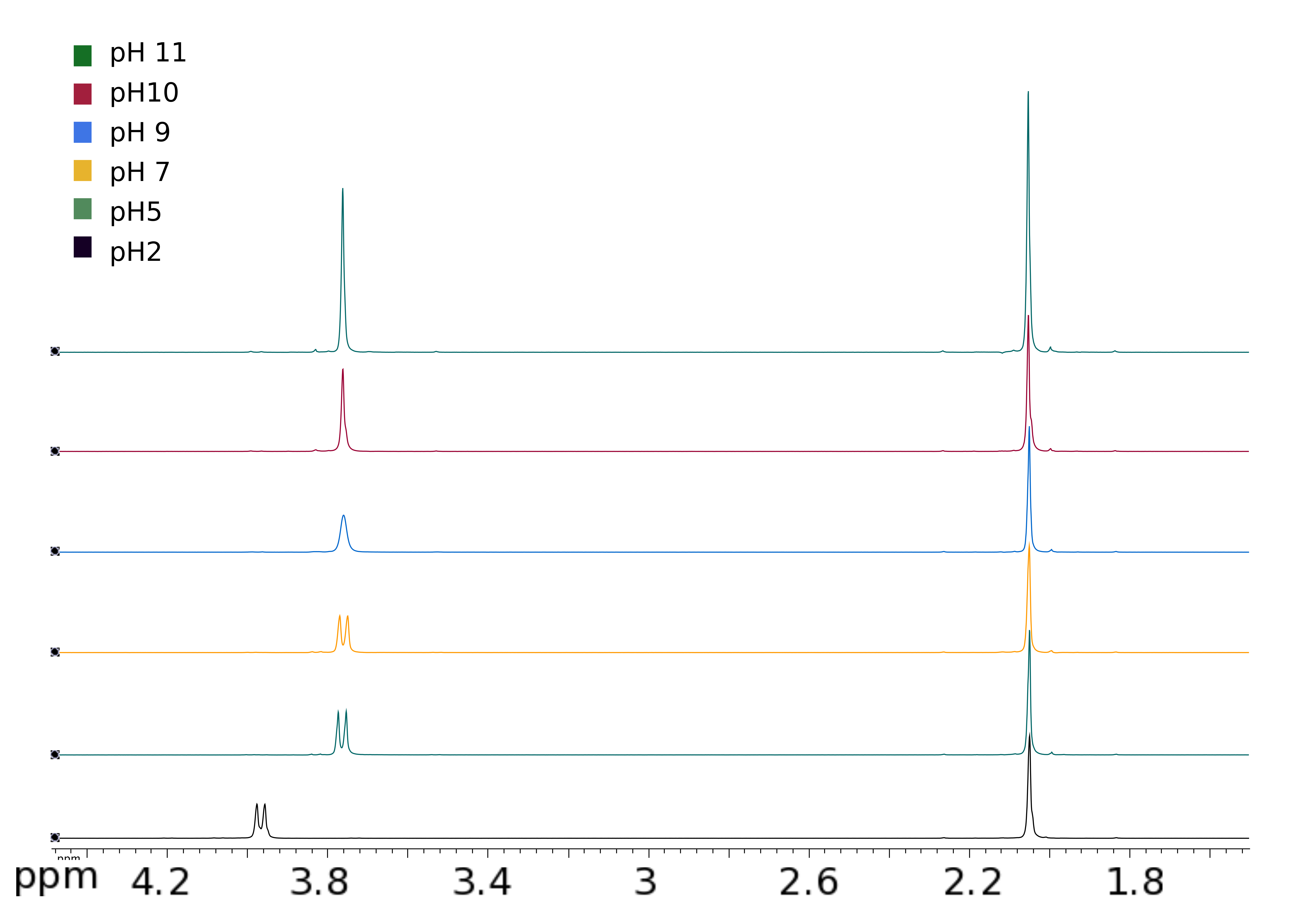

Supplement: Supplementary file 16 — High resolution image (TIFF 748 kb) [file 11084_2018_9555_MOESM8_ESM.tif]

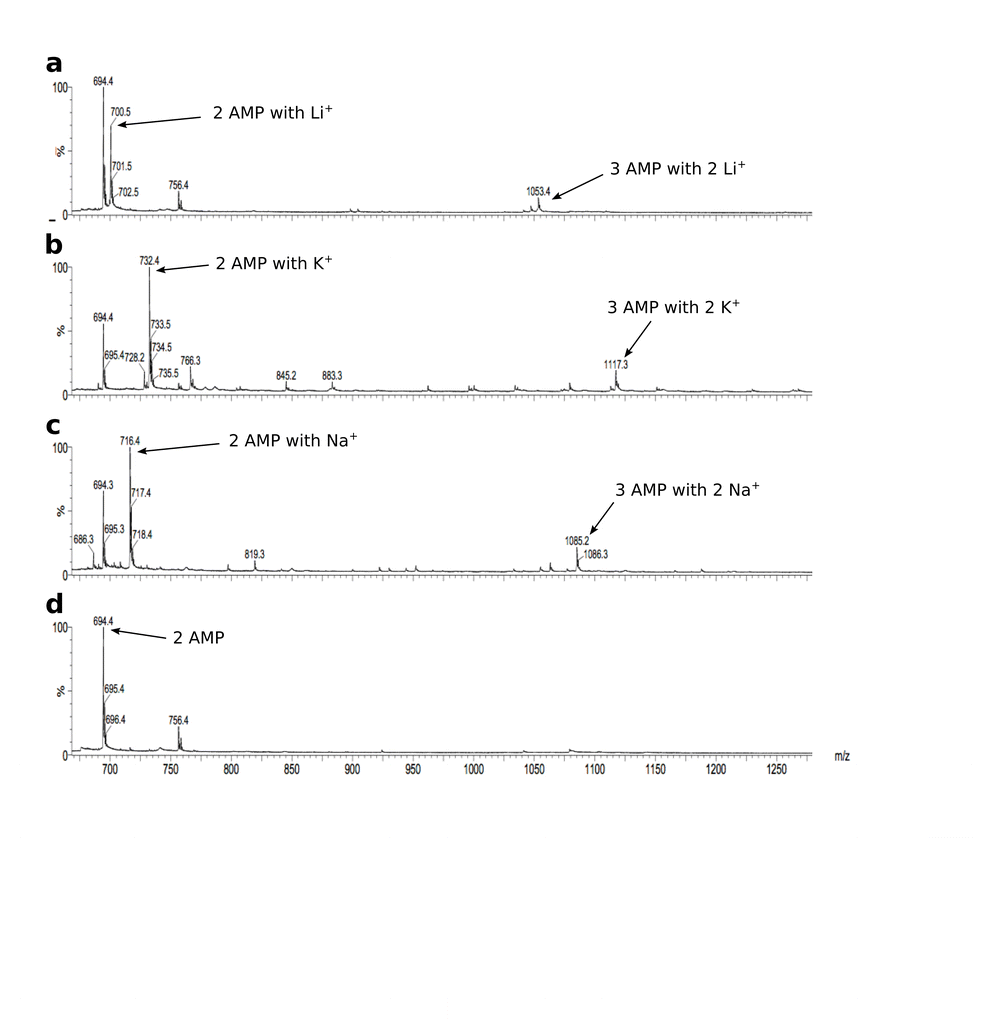

Supplement: Supplementary file 17 — MALDI-TOF mass spectra of solutions of 3 mM AMP with added (a) 3 mM LiCl, (b) 3 mM KOH, (c) 3 mM NaOH and (d) water. Main peaks corresponding to stacks of AMP molecules with each cation are shown for clarity. The size of an AMP monomer is of 347.22 m/z. (GIF 47 kb) [file 11084_2018_9555_Fig16_ESM.gif]

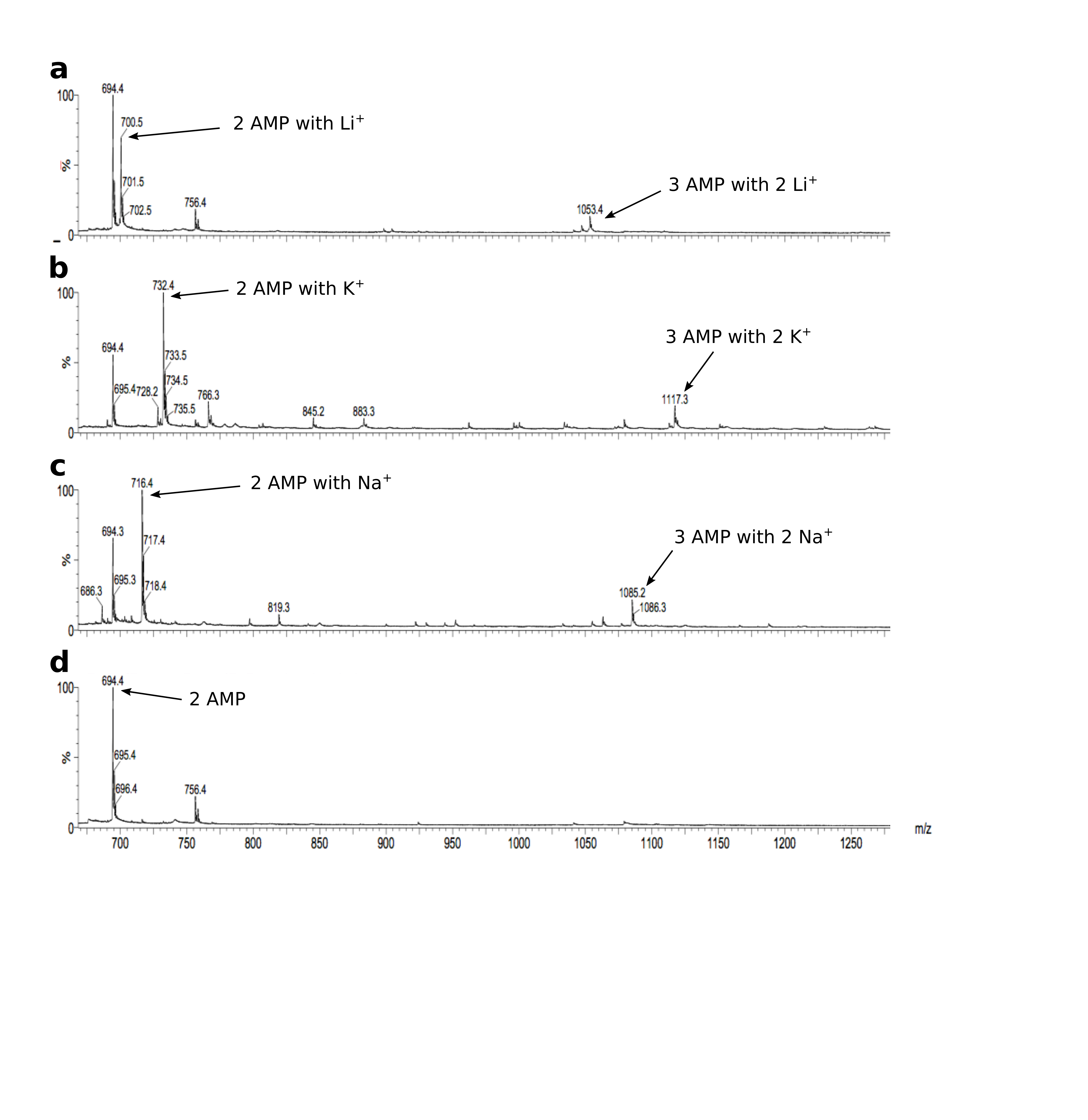

Supplement: Supplementary file 18 — High resolution image (TIFF 2409 kb) [file 11084_2018_9555_MOESM9_ESM.tif]
